# Advances in high-performance MEMS pressure sensors: design, fabrication, and packaging

**DOI:** 10.1038/s41378-023-00620-1

**Published:** 2023-12-19

**Authors:** Xiangguang Han, Mimi Huang, Zutang Wu, Yi Gao, Yong Xia, Ping Yang, Shu Fan, Xuhao Lu, Xiaokai Yang, Lin Liang, Wenbi Su, Lu Wang, Zeyu Cui, Yihe Zhao, Zhikang Li, Libo Zhao, Zhuangde Jiang

**Affiliations:** 1 State Key Laboratory for Manufacturing Systems Engineering, Xi’an Jiaotong University, Xi’an, 710049 China; 2 International Joint Laboratory for Micro/Nano Manufacturing and Measurement Technologies, Xi’an Jiaotong University, Xi’an, 710049 China; 3 School of Mechanical Engineering, Xi’an Jiaotong University, Xi’an, 710049 China; 4 Northwest Institute of Nuclear Technology, Xi’an, 710024 China

**Keywords:** Electrical and electronic engineering, Electronic properties and materials

## Abstract

Pressure sensors play a vital role in aerospace, automotive, medical, and consumer electronics. Although microelectromechanical system (MEMS)-based pressure sensors have been widely used for decades, new trends in pressure sensors, including higher sensitivity, higher accuracy, better multifunctionality, smaller chip size, and smaller package size, have recently emerged. The demand for performance upgradation has led to breakthroughs in sensor materials, design, fabrication, and packaging methods, which have emerged frequently in recent decades. This paper reviews common new trends in MEMS pressure sensors, including minute differential pressure sensors (MDPSs), resonant pressure sensors (RPSs), integrated pressure sensors, miniaturized pressure chips, and leadless pressure sensors. To realize an extremely sensitive MDPS with broad application potential, including in medical ventilators and fire residual pressure monitors, the “beam-membrane-island” sensor design exhibits the best performance of 66 μV/V/kPa with a natural frequency of 11.3 kHz. In high-accuracy applications, silicon and quartz RPS are analyzed, and both materials show ±0.01%FS accuracy with respect to varying temperature coefficient of frequency (*TCF*) control methods. To improve MEMS sensor integration, different integrated “pressure + *x*” sensor designs and fabrication methods are compared. In this realm, the intercoupling effect still requires further investigation. Typical fabrication methods for microsized pressure sensor chips are also reviewed. To date, the chip thickness size can be controlled to be <0.1 mm, which is advantageous for implant sensors. Furthermore, a leadless pressure sensor was analyzed, offering an extremely small package size and harsh environmental compatibility. This review is structured as follows. The background of pressure sensors is first presented. Then, an in-depth introduction to MEMS pressure sensors based on different application scenarios is provided. Additionally, their respective characteristics and significant advancements are analyzed and summarized. Finally, development trends of MEMS pressure sensors in different fields are analyzed.

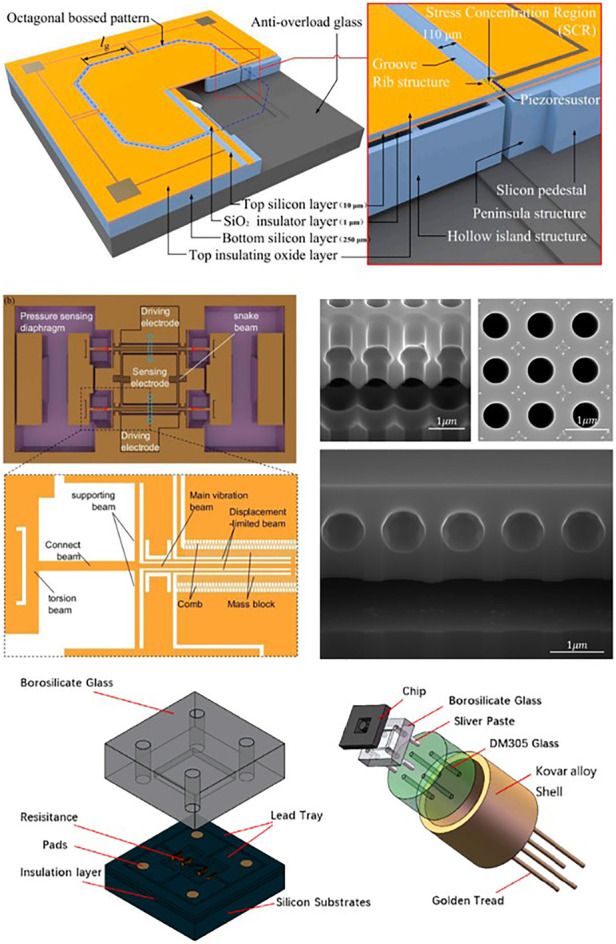

## Introduction

Pressure is a fundamental parameter for measuring internal fluid pressure and is crucial for fluid control and equipment status monitoring. Pressure sensors thus find extensive applications in industries such as automotive, medical, and aerospace. Through precise pressure measurements, equipment conditions can be accurately monitored, and potential failures can be predicted. Furthermore, with the ongoing advancement in smart instruments over the past few decades, there has been a demand for pressure sensors to satisfy stricter technical requirements. These include higher precision, enhanced environmental adaptability, finer resolution, and smaller chip/package sizes^1–4^.

Pressure sensors can be categorized based on their sensing mechanisms into piezoresistive, capacitive, resonant, piezoelectric sensors, and more^5–8^. The piezoresistive effect, which was discovered by Smith in 1954^9^, has led to the widespread use of piezoresistive sensors^10,11^. These sensors primarily measure pressure using a Wheatstone bridge. A pivotal step in chip fabrication that has catalyzed the mass production of piezoresistive pressure sensors (PPS) is diaphragm etching^12,13^. This process is bifurcated into wet and dry etching. Wet etching is further differentiated into anisotropic and isotropic methods. The deep reactive ion etching (DRIE) method, which was introduced in 1993^14^, minimizes lateral etching by alternating between passivation and etching phases^15^. This innovation led to a marked reduction in chip size and cost. However, piezoresistive sensors exhibit a high temperature coefficient of zero point (*TCZ*) and a high temperature coefficient of sensitivity (*TCS*)^16^, diminishing their accuracy across extensive temperature ranges^17,18^. Bao demonstrated that the *TCS* can be offset by balancing the temperature coefficient of resistance (*TCR*) and temperature coefficient of piezoresistivity (*TCP*)^19^. Factors such as piezoresistor *TCR*, fabrication discrepancies, and package stress add complexity to realizing high-precision pressure measurements^20^. Currently, only a handful of companies have successfully commercialized high-precision single-crystal silicon (SCS) pressure sensors. Thus, high-performance PPS still needs further research.

In addition to SCS pressure sensors, a polycrystalline silicon pressure sensor (PSPS) was proposed by French and Evans in 1985^21^. This sensor was used to comprehensively examine the piezoresistive effect between monocrystalline silicon and the barrier region. To describe the theoretical model of the sensitivity coefficient of polycrystalline silicon pressure sensors more accurately under different doping concentrations, Chuai et al. established a tunnel piezoresistive theory in 2012^22^. Currently, PSPS is seldom mass-produced, and its accuracy needs to be further improved^23,24^. As another important piezoresistive pressure sensor, thin-film pressure sensors are based on the strain effect of metals and are widely used in the automotive electronics and aerospace fields, especially as ultralow-temperature pressure sensors. However, the fabrication cost is excessively high for batch consumer electronic applications. The sensing material system also requires further expansion because the sensing unit is mainly concentrated in Ni/Cr material systems^25–28^ with a limited gauge factor.

In addition to PPS, capacitive pressure sensors (CPS) measure pressure chiefly by monitoring the capacitance change between their sensing plates. Notably, CPSs consume significantly less power when compared to piezoresistive or resonant pressure sensors (RPSs)^29^, making them popular choices for consumer electronics and industrial applications. An advantage of CPSs is their improved temperature stability, as their capacitance is less affected by temperature^30^. However, CPSs are more susceptible to variations in media dielectric properties, such as the parasitic capacitance from measurement circuits or the pressure media^31–33^. This limitation can negatively impact their precision. Consequently, only a few CPSs have reported precision values exceeding 0.05%FS. Moreover, some RPSs operate by recording the change in a resonant beam’s stress state due to pressure. This pressure change results in a linear shift of the beam’s natural frequency. Due to their high gauge factor, high-quality factor, and reliance on the elastic modulus to determine output frequency, RPSs offer exceptional detection resolution and accuracy, making them ideal for metrological sensors. However, their intricate structure and fabrication process increase their cost. Moreover, their response frequency is limited by factors such as phase-locked loops and packaging techniques, rendering them less suitable for high-response pressure detection^7,8,20^.

Microelectromechanical system (MEMS) pressure sensors offer several advantages, including ease of batch production, miniaturization, cost-effectiveness, and the capability to readily fabricate complex structures. Therefore, they occupy an increasingly larger share of the pressure sensor market. Over the past two decades, advancements in MEMS sensor technology, encompassing principles, theory, design, and fabrication techniques (such as bulk silicon etching, thin-film fabrication, and low-temperature bonding), have facilitated the rapid advancement and diversification of pressure sensors for various applications. Newer pressure-sensing elements leveraging innovative technologies and packaging methods offer enhanced measurement accuracy, size, and broader temperature adaptability^34,35^. Furthermore, the integration of emerging materials, such as third-generation semiconductor materials, graphene, and nanowires, in pressure sensors has significantly elevated their performance^36,37^.

Relevant MEMS pressure sensor trends include drives for higher sensitivity, higher accuracy, multiparameter integration, smaller chip size, smaller package size, and harsh environment-compatible devices. This paper reviews the corresponding sensors in various applications, including minute differential pressure sensors (MDPSs), RPSs, integrated pressure sensors, miniaturized pressure sensors, and leadless packaged pressure sensors. The working principles, research progress, technical difficulties, and prospects are discussed separately.

## Minute differential pressure sensors

MDPSs have found applications in diverse fields, including biomedicine^38,39^, aerospace^40^, blast damage assessment^41^, and air pressure monitoring in fire exits^42,43^, underscoring their significant engineering and medical value. An MDPS generally refers to a pressure sensor capable of measuring pressure ranges below 10 kPa. This indicates that the differential pressure across the diaphragm is usually minimal. As medical equipment evolves, there is a growing demand for MDPSs with superior measurement resolution, enhanced frequency response, reduced size, and cost-effectiveness. Designing and fabricating MDPSs present greater challenges when compared to other piezoresistive sensors, especially in ensuring high sensitivity and embedding a stable piezoresistor on an ultrathin, low-residual-stress pressure diaphragm. Therefore, over the past 20 years, many researchers have conducted in-depth studies on MDPSs based on their principles, materials, and structures.

MDPSs typically measure pressure ranges spanning just a few kPa or even down to hundreds of Pa. Consequently, they demand high sensitivity and resolution, leading to sensing diaphragms that often possess a high width-to-thickness ratio. For capacitive MDPSs, their large outer diameter and pronounced nonlinearity limit their application in minute pressure sensing^44,45^. However, piezoresistive pressure sensors exhibit favorable linearity and capitalize on the stress concentration effect of the sensing unit. This feature makes them conducive to chip miniaturization, explaining their extensive investigation in the literature^46^. To satisfy the sensitivity requirements of MDPSs, researchers introduced a variety of sensitive structures for ultrasensitive MEMS sensors. Over time, MDPSs have evolved from “C”-type membranes^5^ (flat membranes) to “E”-type membranes^47^ (island membranes) and then to “beam–membrane–island” configurations^48,49^, witnessing progressive enhancements in sensitivity.

**Fig. 1:**
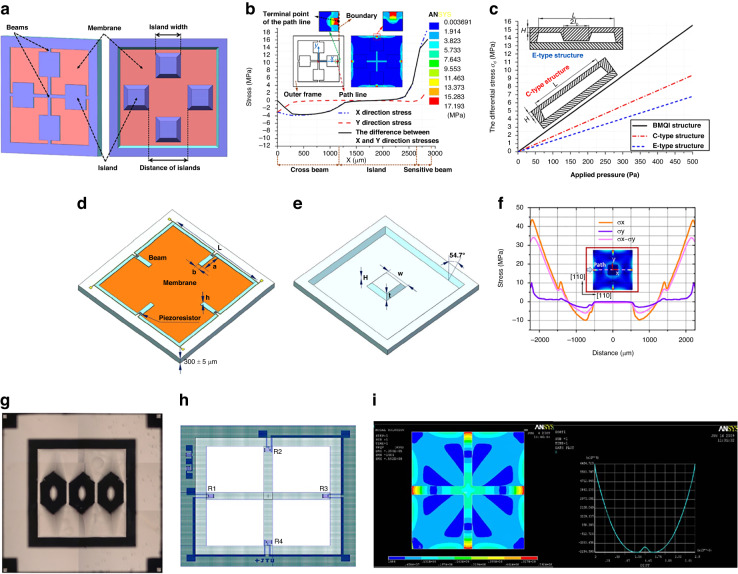
MDPS design. **a** MDPS characterized by a “beam-membrane-quad-island” (BMQI) structure (front and bottom view of the chip)^47^. **b** Stress distribution and stress path of the structure. **c** Comparison of the von Mises stresses of the three structure types. **d** Four-beam-bossed-membrane (FBBM) structure (front and back review)^50^. **e** Back review of FBBM^50^. **f** Stress distribution curve of the path^50^. **g** Backside view of an ultrahigh-sensitivity pressure sensor chip^51^. **h** Circuit on the CBM sensor. **i** Structure of CBM and X-direction stress distribution curve^52^

To improve the sensitivity of MDPSs, a sensitive resistor is set in the stress concentration area to eliminate unnecessary energy loss. Yu et al. ^47^ combined a surface cross-beam and backside islands based on a C-type membrane structure (Fig. 1a). The design combination further localizes the stress concentration on the beams near the diaphragm edge, as illustrated in Fig. 1b. This design results in a sensitivity output that surpasses both the C-type and E-type structures, as depicted in Fig. 1c. Additionally, with the inclusion of the rectangular silicon island limiter, the diaphragm can endure atmospheric pressures up to 200 times its pressure range. The results show that the structure can significantly improve the sensitivity and reach 11.098 μV/V/Pa in the pressure range of 0–500 Pa. However, the nonlinearity is only 3.046%FS.

Li et al. ^50^ enhanced the stress concentration effect using a crossbeam film and introduced a high-sensitivity structure by integrating the film with an annular groove, as illustrated in Fig. 1d, e. In this configuration, the crossbeam serves as a supporting rib, preventing undue deformation. Four grooves are etched adjacent to the membrane, inducing a rapid change in the lateral stiffness of the sensing area and amplifying the stress concentration to 40 MPa, as demonstrated in Fig. 1f. This design exhibits a sensitivity of 30.9 mV/V/kPa within a pressure range of 145 Pa and maintains a nonlinearity of just 0.25% FS. However, the design presents a sizable chip dimension of 7 mm × 7 mm. Basov et al. ^51^, in their pursuit of heightened sensitivity, created MDPS islands using wet etching, incorporating a “multi-island” design for stress concentration, as depicted in Fig. 1g. The concentration is determined by the spacing between the islands. After optimization, the sensitivity reached an impressive 34.5 mV/V/kPa, with nonlinearity surpassing 0.81% FS within a 500 Pa pressure scope. However, the chip measures 6.15 mm × 6.15 mm. Tian et al. ^52^ introduced a cross-beam membrane (CBM), showcased in Fig. 1h, which boasts superior stress concentration capabilities. It delivers an accuracy of ~0.24%FS within a 10 kPa pressure range, with a chip size of 4.3 mm × 4.3 mm.

To satisfy the requirements for monitoring infant respiratory pressure and pipette height, there is a compelling need to boost sensor sensitivity. Xu advanced the “beam–island–membrane” structure by incorporating the “peninsula island” design, which amplified the stress concentration effect^48,49,53^, as illustrated in Fig. 3a, b. Additionally, shallow grooves were etched at the forefront of the pressure-bearing diaphragm. The ridges that form between these grooves cause a pronounced shift in lateral stiffness, preventing strain energy dispersion and amassing stress >50 MPa, as depicted in Fig. 3c. The innovative hollow island design curtails excessive diaphragm mass, enhancing the sensor’s dynamic performance. This configuration yields an impressive sensitivity of 66 μV/V/kPa and maintains a nonlinearity of 0.33%FS within a 500 Pa range.

**Fig. 2:**
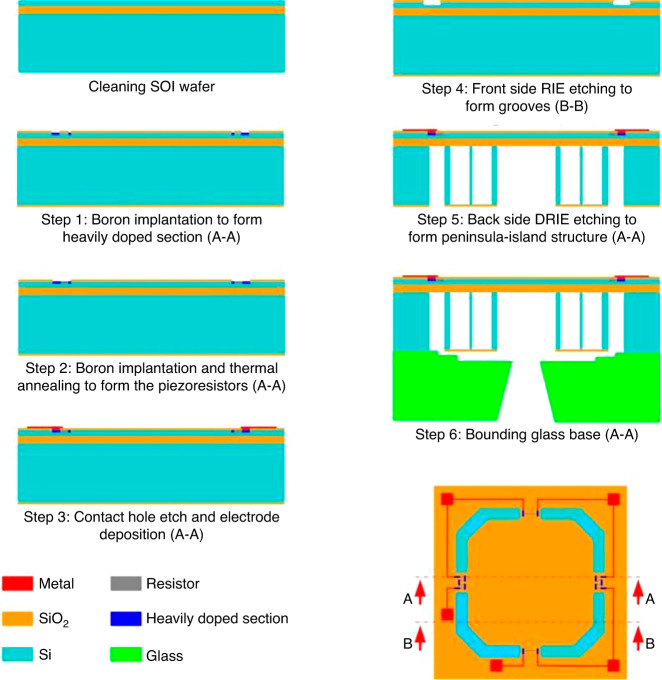
Fabrication process of a typical MDPS^39^. **a** Process flow, **a-1** Cleaning SOI wafer, **a-2** Boron implantation to form heavily doped section, **a-3** Boron implantation and thermal annealing to form the piezoresistors, **a-4** Contact hole etching, **a-5** Front side RIE etching to form grooves, **a-6** Back side DRIE etching to from peninsula-island structure, **a-7** Bonding glass base, **b** Top view of the typical MDPS

Different MDPS pressure sensors adopt similar fabrication processes, including sensitive piezoresistor doping and wet/dry etching, as shown in Fig. 2. In most designs, an etching-stop layer is introduced for thickness control, which is difficult to realize using single-crystal silicon direct etching. Given that the diaphragm is too thin to be sensitive to its stress state, there is a lack of stress control methods. Thus, it should be investigated in the future.

**Fig. 3:**
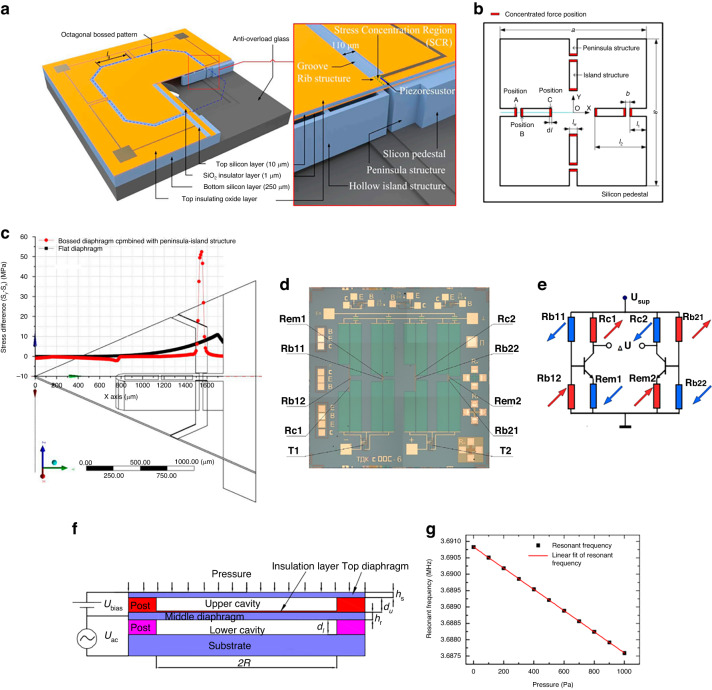
High-sensitivity MDPS, on-chip amplified MDPS, and resonant MDPS. **a** Peninsula island-based bossed diaphragm structure^48,49,53^. **b** Planar construction view of the proposed structure^48,49,53^. **c** Stress difference from the central point to the diaphragm edge^48,49,53^. **d** Ultrahigh-sensitivity pressure sensor (top view),^185^. **e** Electrical circuit^185^. **f** Structural schematic of the improved capacitive resonant MDPS^55^. **g** Variation in the resonant frequency of the transducer with pressure^55^

In addition to driving modified structural design, signal amplification can increase pressure sensitivity and resolution. Basov et al. ^54^ proposed a piezoresistive differential amplifier using an on-chip negative-feedback loop. The combination design with the on-chip circuit amplifier and stress concentration structure is shown in Fig. 3d, and a schematic of the electrical circuit is shown in Fig. 3e. After amplification, the MDPS sensitivity reaches 44.9 mV/V/kPa, and the *TCZ* is ~0.094% FS/°C with a 550-fold overload ability.

In addition to piezoresistive sensors, miniature capacitive resonant pressure sensors (RPSs) have been developed with high accuracy and resolution. Li et al. ^55^ developed a resonant pressure sensor in which the pressure diaphragm stress changes under pressure, causing the stiffness of the diaphragm to shift linearly, and the frequency reflects the pressure, as shown in Fig. 3f. The test results are shown in Fig. 3g. The frequency pressure sensitivity is ~−2.54 ppm/Pa (7.46 Hz/Pa); the nonlinearity is <0.01%FS, which is much better than that of the piezoresistive sensor. However, this capacitive RPS requires more complicated conditioning circuits and relies on the pressure medium density. Thus, its applications are highly limited.

The specifications of the reported MDPSs are listed in Table 1. The “slot + island + peninsula” structure has higher sensor sensitivity. However, with a large in-plane size of 5 mm × 5 mm, the size of the sensor chip should be further reduced to satisfy the requirements for miniaturized packaging. Moreover, research on MDPSs is currently mainly focused on improving the sensitivity, which nearly satisfies the resolution requirements of common medical equipment. In many applications, such as shockwaves and altimeters in flight control, the dynamic characteristics of the sensor are needed. Thus, a decoupling method for sensitivity and frequency should be investigated. Additionally, the nonlinearity of the reported sensor still requires improvement, and the stress of the multilayer diaphragm at different temperatures should be studied to realize such improvement because the diaphragm is usually thin and sensitive to residual stress. The resonant-capacitance MDPS exhibits better accuracy than other designs. However, it requires a complex control circuit.

**Table 1:** Performance comparisons of typical reported MDPSs in previous studies

Author	Pressure	Structure	Sensitivity	Nonlinearity	Frequency	Chip size
Xu^53^	500 Pa	Slot + island + peninsula	0.066 mV/V/kPa	0.33%FS	11.3 kHz	5 mm × 5 mm
Yu^47^	500 Pa	Beam–island	11.089 µV/V/Pa	3.046%	6.9 kHz	7 mm × 7 mm
Li^50^	145 Pa	Slot+beam	30.9 mV/V/kPa	0.25%FS	\	6 mm × 6 mm
Mikhail^51^	500 Pa	Multi-island	34.5 mV/V/kPa	0.81%FS	\	6 mm × 6 mm
Li^55^	1000 Pa	Resonant	7.46 Hz/Pa	0.01% FS	\	\

## RPS

High-precision pressure sensors are critically important in sectors such as aerospace, oil exploration, meteorological observation, and national defense. They are often employed to measure fluid pressures in ambient environments, cabin atmospheres, fuselage hydraulic systems, engines, and the gases and liquids of oxygen masks^40,56^. Within aviation’s air data detection systems, an integrated data system typically necessitates over ten high-precision pressure sensors. These sensors are expected to achieve accuracy surpassing ±0.05%FS and ensure long-term stability in their performance^57–59^. High-precision pressure sensors play an irreplaceable role in the fields of meteorological measurements and air data systems. The accuracy is generally required to be an order of magnitude higher than that of the currently available sensors, and the basic error is usually expected to be <0.02%FS^10^.

Currently, the global landscape of high-precision pressure sensors predominantly features RPS with an extensive operational temperature range. Moreover, only a few piezoresistive sensors can realize an accuracy of 0.05%FS^60–62^. However, they often exhibit inferior temperature stability. Over recent decades, extensive studies on RPSs have delved into their sensitive materials, structures, packaging solutions, and methods of excitation and detection. This discussion will explore the current research on RPSs, particularly focusing on their excitation and detection methods. The *Q*-factor is a pivotal metric for RPS, which indicates the resolution ability to detect resonant frequencies. The pressure resolution is essentially contingent upon the *Q*-factor. Numerous efforts have aimed to elevate the *Q*-factor by mitigating sources of damping, such as air and thermoelastic damping.

### Piezoelectric RPS

Piezoelectric conversion serves dual purposes: it detects mechanical deformation via the piezoelectric effect and excites mechanical structures to induce vibrations via the inverse piezoelectric effect. The latter purpose is particularly employed to stimulate the resonant beam, with the stimulation directed along a specific crystal axis of the piezoelectric material^63^. Most reported piezoelectric resonant devices utilize quartz crystals, renowned for their piezoelectric effects, or thin-layer Al_5_N_3_ produced via magnetron sputtering. Alternatives, such as ZnO or PZT, are less congruent with semiconductor technology. Quartz piezoelectric crystal materials, known for their minimal hysteresis, high *Q*-factor, and commendable temperature stability^64^, have been extensively adopted in crystal oscillators. Thus, they emerge as a prime material choice for RPS fabrication. In quartz RPS designs, the quartz crystal beam concurrently operates as the driver and detector.

**Fig. 4:**
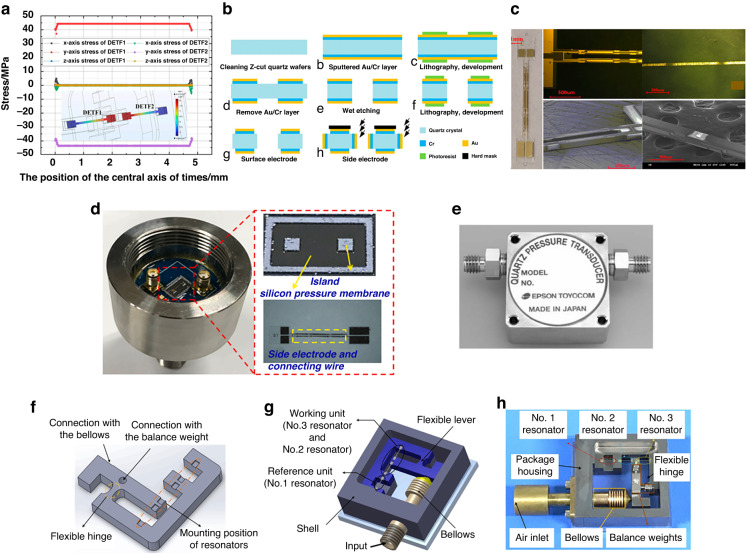
Quartz RPSs. **a** Stress simulation of differential output tuning fork tines^65^. **b** Main fabrication process of the DETF quartz resonator^65^. **c** Photograph of the tuning fork prototype^65^. **d** Packaged pressure sensor prototype with a tuning fork and stainless-steel package^68^^,^^69^. **e** Photograph of the EPSON quartz pressure sensor^69^. **f** Overall structural diagram of a flexible hinge lever^70^. **g** Main structural diagram of the sensor^70^. **h** Photograph of the sensor before packaging^70^

Zhao^65–67^ introduced a range of differential RPSs utilizing piezoelectric quartz resonant beams. The resonator undergoes fabrication through anisotropic etching, and the quartz sensing component is affixed to a silicon membrane, as depicted in Fig. 4a. Using side-magnetron sputtering, excitation and detection electrodes are produced, as illustrated in Fig. 4b. Testing revealed an accuracy surpassing 0.05% FS within the range of 10 kPa. To mitigate temperature sensitivity, dual differential resonators were integrated to compensate for temperature and stress. Nonetheless, the mismatch in the coefficient of thermal expansion (*CTE*) between the quartz resonator and silicon diaphragm results in significant thermal stress, compromising accuracy. The in-plane dimension of the quartz resonant sensor chip measured ~8 mm, supporting that the size can be further reduced, as indicated in Fig. 4c.

To address challenges associated with assembling quartz crystal sensing elements and pressure transfer structures, researchers combined traditional machining with MEMS processes. Specifically, wet etching is used for shaping the quartz structure, while traditional precision machining helps to realize the pressure conversion element. The quartz RPS designed by Ren^68^ (depicted in Fig. 4d) and the Epson Company^69^ (illustrated in Fig. 4e) feature a metal base and tuning fork resonator. They offer a repeatability of 0.005%FS and hysteresis of 0.008%FS. Additionally, Zhang et al. ^70^ integrated a mechanical lever structure (Fig. 4f) with three resonators, as represented in Fig. 4g and h. This device realized a high precision of ±0.01% FS over a range of 100 kPa. However, this type of packaging approach demands exact machining and assembly standards. Coupled with the size and intricacy of the package, the resultant costs are elevated. Therefore, further refinements to shrink the design of quartz RPSs are warranted.

### Capacitive resonant pressure sensor

In addition to piezoelectric RPSs, breakthroughs in silicon-based RPSs, including capacitive, piezoresistive, and magnetic RPSs, have been realized in recent years. Electrostatic excitation is currently the most widely used excitation method for resonant sensors such as resonant pressure, acceleration, and gyroscopes^71–73^. Electrostatic excitation refers to the use of an electrostatic force between the driving and fixed plates by applying an AC + DC voltage with a driving frequency similar to the resonant frequency. Capacitance detection identifies the resonant frequency point of the resonator by detecting the capacitance changes during vibrations. Capacitance RPS has a simpler fabrication process than piezoelectric, piezoresistive, and other preparation processes, including etching and bonding, and thereby has attracted the attention of many researchers.

**Fig. 5:**
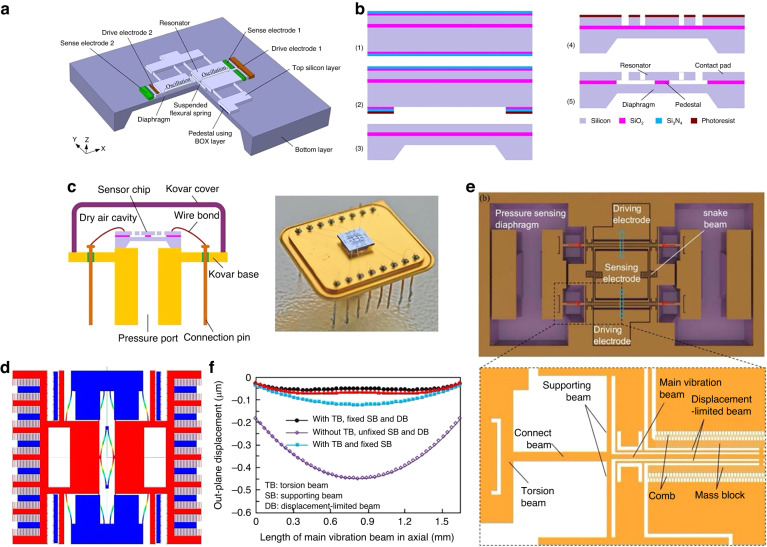
Capacitive RPS. **a** Schematic of RPS proposed by Ren^74,75^. **b** Fabrication process^74,75^. **c** RPS package with transistor outline (TO) base and Kovar Cover^74,75^. **d** New tuning fork structure for the RPS. **e** Design structure of the balanced double-ended tuning fork (BDETF) resonator^76,78^. **f** Comparison of out-of-plane displacements for different beam structures^76,78^

Ren et al. proposed an RPS based on a double-ended fixed-branch tuning fork structure^74,75^, as shown in Fig. 5a. This design used SOI release technology to realize the suspension of the resonator structure, and a nonmetal pad was introduced because most metals are incompatible with the HF solution, as shown in Fig. 5b. In the no-pad bonding technique, the gold wire bonds directly to the silicon surface, which can easily lead to detachment and compromise the sensor’s reliability. When compared to the fabrication technique that employs wet etching and fusion bonding of silicon islands, this challenge is considerably reduced. Testing indicates a sensitivity of ~10.86 Hz/kPa, with a basic measurement error of ±0.02%FS. Nonetheless, the sensor’s quality factor was low, as the resonator was not vacuum-packaged, as depicted in Fig. 5c. Furthermore, given the minimal Δ*C* during vibration, Sun^75^ introduced a new expanded capacitance driving and detection structure. This structure was realized by altering the interface area relative to the electrode distance, as illustrated in Fig. 5d, which eases signal detection challenges.

For capacitance excitation, it is crucial to maintain a consistent driving force across the full pressure range, particularly for closed-loop control. Recognizing the significant out-of-plane displacement discrepancy between the fixed and movable electrodes of the capacitive detection RPS under stress, Du et al. ^76–78^ introduced a dual-diaphragm RPS design, depicted in Fig. 5e. This design utilizes a composite beam structure, which considerably reduces the out-of-plane displacement of the resonator’s movable electrode, as illustrated in Fig. 5f. Leveraging the anodic bonding auxiliary getter process, they realized high-vacuum packaging. Consequently, the quality factor surpassed 20,000, with the sensor sensitivity reaching 30 Hz/kPa.

In capacitance-based RPS systems, the change in capacitance Δ*C*, which is rooted in interdigital capacitance, is typically minuscule—often at the fF scale. Thus, it is challenging to detect and often necessitates a complex CV conversion circuit with expansive gain and frequency response. Furthermore, significant crosstalk can arise between the excitation and detection electrodes, leading to pronounced interference in closed-loop control. To address this issue, Du^76^ implemented a single pickup electrode situated between the two resonating beams. With this arrangement, during symmetrical resonator vibration, the capacitor functions as expected. Conversely, when there is a unilateral resonator vibration, the capacitance variations from the two pickup electrodes neutralize each other. This design essentially shields the same-side vibration mode.

### Electromagnetic RPS

An RPS utilizing electromagnetic excitation/detection mainly leverages the alternating Lorentz force. This force is produced by the alternating current acting on a resonant beam within a constant magnetic field, prompting the resonant beam to oscillate. When the resonator achieves a state of resonance, the oscillating detection beam intersects the magnetic field lines, and the resulting electromagnetic induction generates a signal that marks the resonance frequency point. Unlike the previously discussed resonant sensors, an electromagnetic device does not require intricate excitation or pickup structures. It simply demands an alternating current to be channeled through the driving beam and AC signal detection on the detecting beam. This simplicity in design and manufacturing has catalyzed further research and successful commercialization^79,80^.

**Fig. 6:**
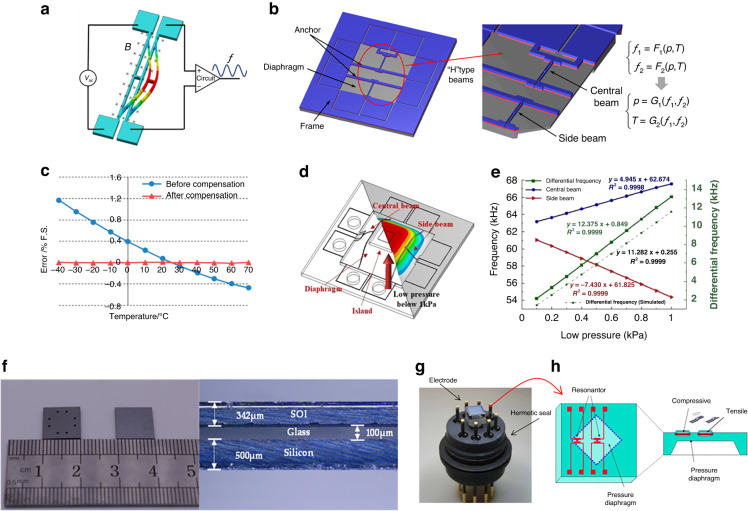
RPS based on the electromagnetic method. **a** Resonator based on electromagnetic excitation and detection, where the first resonant mode is adopted as the working mode^81,82^. **b** Schematic of the differential pressure sensor with double “H”-type double-clamped resonant beams^81,82^. **c** Comparison of errors before and after differential compensation^81,82^. **d** Schematic of the developed micromachined resonant low-pressure sensor. **e** Intrinsic frequency shifts of the central (blue) and side (red) beams and differential outputs (green) as functions of low pressure at room temperature. **f** Front/back views of the microsensor chip, side view of the microsensor chip and layer-by-layer view of the core pressure-sensitive element with a stress isolation layer. **g** Differential RPS developed by Yokawaga^84–86^. **h** Cross-sectional view of the differential RPS chip

Wang et al. ^81–83^ pioneered various RPSs utilizing electromagnetic techniques. To enhance the sensor’s temperature stability, they employed a differential frequency output method, as depicted in Fig. 6b. This method incorporates two or three resonators to decouple the pressure and temperature signals, subsequently reducing the *TCF*, as illustrated in Fig. 6c. The resonator comprises “H”-type resonant beams with a single-sided vibration mode chosen as the operational mode, as shown in Fig. 6a. To guarantee a high-quality factor, they leveraged anodic bonding technology and a getter process to ensure vacuum packaging. In addition to the differential approach, a unique stress-isolation structure was introduced to counteract the package stress, thereby further minimizing the *TCF*. Within a pressure range of 100 kPa, the sensor showed a sensitivity of 89.86 Hz/kPa, resolution of 10 Pa, and nonlinearity of <0.01% FS. Additionally, for high-precision MDPS applications, they integrated the “island–diaphragm–beam” design to enhance RPS sensitivity, as evident in Fig. 6d. Their findings demonstrated accuracy surpassing 0.05%FS in a 1-kPa application, as highlighted in Fig. 6e.

High-precision differential pressure RPSs are rarely explored and documented, primarily due to the challenges of negating and compensating for the static pressure on either side of the diaphragm and temperature variations. To address this challenge, Wang^83,84^ introduced a differential RPS incorporating a petite electromagnetic resonator and multilayer-assembled diaphragm, as depicted in Fig. 6f. Similarly, Yokogawa presented a differential RPS tailored for industrial usage based on electromagnetic excitation/detection. This innovation has since been successfully commercialized^85–89^ and is illustrated in Fig. 6g. Their design integrates two sets of “H”-shaped resonant beams. Unlike Wang et al.’s research, they opted for an out-of-plane vibration mode, resulting in a reduced resonant layer thickness. To amplify sensitivity, the resonators were strategically positioned at both the diaphragm’s center and periphery, inducing tension and compression, respectively. This layout, visualized in Fig. 6h, adeptly minimized *TCF*. The ’fabrication of the resonator leveraged epitaxial and anisotropic etching techniques, resulting in a *Q*-factor exceeding 50,000, a *TCF* under 40 ppm/°C, a fundamental error less than ±0.02%FS, and a stability of ±0.1%FS over a decade. Moreover, given that both sides of the diaphragm are exposed to pressure media, the sensor can be used for gauge pressure or differential pressure measurements, making it one of the few reported gauge RPSs.

### Piezoresistive detection RPS

The excitation method for piezoresistive detection is driven by an electrostatic force, which is the same as that for the capacitance detection of RPS. Piezoresistive pick-up resistors are typically arranged in the stress concentration area of the resonant beam, and the resonant frequency is detected by monitoring the change in resistance. Compared to the capacitive detection method, piezoresistive detection has a larger output signal and simpler vibration pickup structure. However, because the piezoresistor fabrication process is more complex than that of the capacitive interdigitated electrode, there is a lack of studies focusing on the piezoresistive detection of RPS^90,91^.

**Fig. 7:**
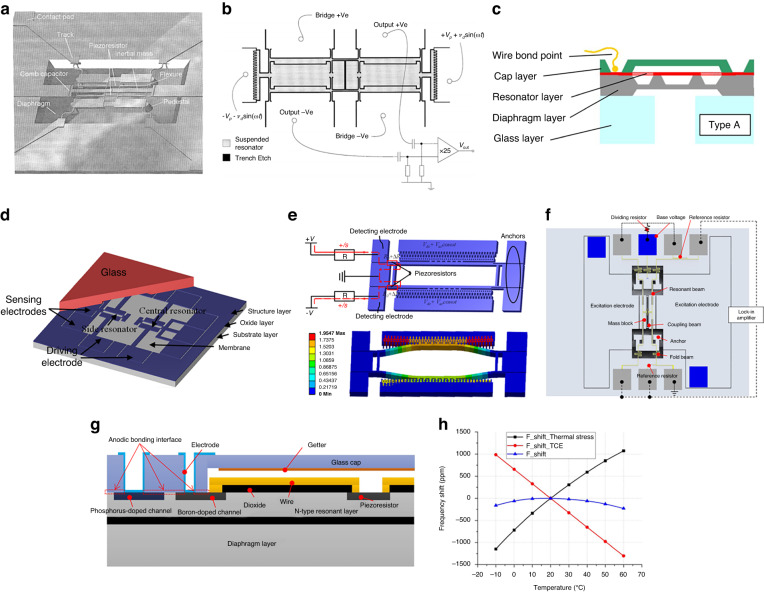
Piezoresistive detection PRS. **a** 3D schematic of the main components of lateral RPS^91,92^. **b** Schematic of electrical connections to the resonator^59,91,92^. **c** Cross-sectional view of the Druck RPS chip^59,91,92^. **d** Schematic of microfabricated differential piezoresistive detection of RPS^93^. **e** Setup and working mode simulation of double-ended tuning forks relying on comb-drive actuation and piezoresistive detection^93^. **f** Schematic of a resonator with a coupling-beam-enhanced DETF^94,96^. **g** Vacuum package schematic of anodic bonding^94,96^. **h** *TCF* self-compensated method with stress and *TCE* contraction^94,96^

In 2009, Druck Co. developed a commercialized RPS based on electrostatic excitation/piezoresistive detection and proposed an electrical connection for the resonator^59,92^ (Fig. 7a, and b). The sensor comprised four layers: a vacuum packaging layer, resonant layer, sensitive film layer, and base glass layer (shown in Fig. 7c). The sensor was mainly prepared via deep reactive ion etching (DRIE) and silicon–silicon bonding, and its *Q*-factor was >30,000. The accuracy is better than 0.01%FS, and the annual stability is better than ±0.01%, setting it as the benchmark product in the high-accuracy RPS industry.

Yan et al. ^93^ also developed a differential RPS based on the piezoresistive detection method, which shielded the signal from adjacent modes, as shown in Fig. 7d; the electrical connection method and working mode are shown in Fig. 7e. This composite beam structure effectively reduced the influence of temperature and packaging stress via differential compensation, and its accuracy reached 0.01% FS. Although this design uses vacuum packaging, its *Q*-factor is only 10,000. Thus, it is difficult to achieve high-resolution measurements. Moreover, the length and width of the RPS are >10 mm, and therefore, it is challenging to realize miniaturization. Han et al. ^94–96^ developed a temperature-self-compensating method using thermal stress to offset the thermal coefficient of the elastic modulus effect, aiming to improve the poor temperature stability of the RPS, as shown in Fig. 7f. A novel vacuum signal transfer method for piezoresistive detection and comb driving is proposed, as shown in Fig. 7g. The *TCF* reduced from the original 32 ppm/°C to the compensated 7.2 ppm/°C, as shown in Fig. 7h. Furthermore, the measurement accuracy reaches 0.02%FS, and the *Q*-factor is >25,000.

### Closed-loop controlled circuit of RPS

A closed-loop control circuit is an essential component of a MEMS resonant pressure sensor (RPS). This design was responsible for maintaining the sensor’s resonant frequency at a predetermined value, which enables accurate pressure measurements^97^. Various techniques, including the frequency locking technique^98,99^, phase-locked loop (PLL) technique^100–104^, adaptive control technique and digital control technique^105,106^, and proportional-integral-derivative (PID) control technique^74,77,94,107^, have been developed to drive mechanical motion, control feedback, and track frequency changes. For the circuit design described above, establishing the equivalent circuit model of an RPS provides an important foundation for sensor characterization and closed-loop circuit design.

**Fig. 8:**
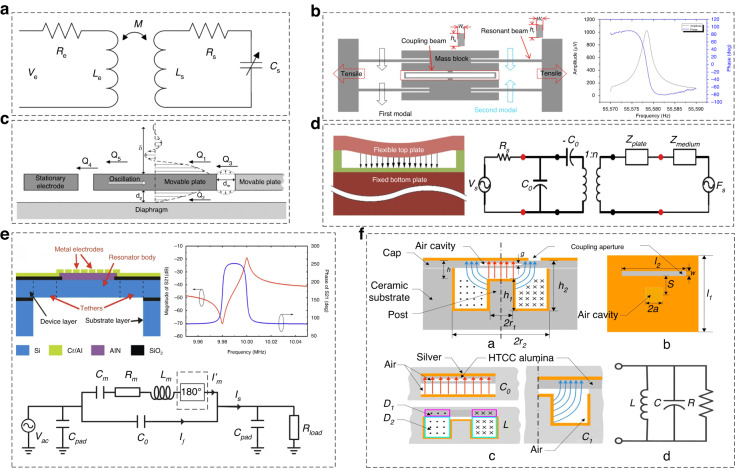
Equivalent circuit model of the resonant pressure sensor. **a** Equivalent circuit of the passive pressure sensor^107^. **b** Typical structure of the resonator and the amplitude-frequency analysis^94^. **c** Damping distribution in the resonant pressure resonator^74^. **d** Axisymmetric cross-section of capacitive pressure transducers and electromechanical coupling model^108^. **e** MEMS squeeze-film pressure sensor and the equivalent circuit model for readout and actuation^109^. **f** Model of a proposed thin-film piezoelectric-on-silicon MEMS resonant pressure sensor^112^

The equivalent circuit model for an RPS provides a simplified representation of its electrical behavior^108,109^. It consists of various electrical components that approximate the mechanical and electrical characteristics of the sensor^110^, as shown in Fig. 8. Understanding this model is crucial for analyzing sensor performance and designing the associated electronic circuitry, especially for closed-loop control circuits^111–116^. The Butterworth-Van-Dyke model is a commonly used lumped-element model composed of four elements: series resistor *R*_m_, series inductance *L*_m_, series capacitance *C*_m_, and parallel capacitance *C*_0_^114^. The resistance component *R*_m_ represents the energy dissipation within the sensor due to various sources, including mechanical losses, damping effects, and electrical losses in the conductive paths. The inductance component, *L*_m_, represents the equivalent mass. This inductance primarily arises from the mechanical motion of the vibrating diaphragm of the sensor and its interaction with the surrounding magnetic field. The series capacitance *C*_m_ represents the equivalent stiffness of the RPS. This accounts for the capacitance between the electrodes of the sensor, which typically results from an overlap between the stationary and moving parts of the MEMS structure. The parallel capacitance *C*_0_ indicates the physical capacitance.

When an excitation voltage is applied to the RPS to induce resonance and initiate mechanical vibrations, the current flowing through the equivalent circuit represents the electrical response of the sensor to an applied excitation voltage^113^. The excitation voltage can be a sinusoidal signal or any other appropriate excitation waveform. Various performance characteristics of the resonant pressure sensor can be examined by analyzing the equivalent circuit model. For example, the impedance response of a circuit can provide insight into the resonant frequency and *Q*-factor of the RPS. The *Q*-factor represents the sharpness of the resonance and is related to the sensitivity and bandwidth of the sensor. The specific components and their values in the equivalent circuit model can vary depending on the design and technology used in the resonant pressure sensor. The model can also be expanded to include additional elements to capture more complex behaviors or account for the specific characteristics of the sensor. The design of the associated electronic circuitry for a resonant pressure sensor, such as the closed-loop control circuit discussed previously, often involves considering an equivalent circuit model. The desired electrical response can be achieved by appropriately selecting the components and configuring the circuit, enabling accurate pressure measurements and control.

**Fig. 9:**
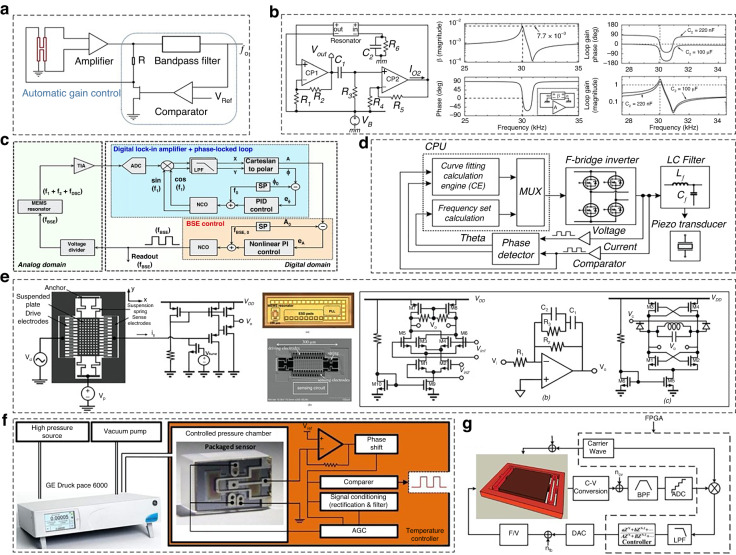
Closed-loop control circuit techniques for RPS. **a** Schematic of the closed-loop circuit with automatic gain control^100^. **b** Schematic view of the oscillator and the frequency response of the loop gain^101^. **c** Digital control system for frequency signals^98^. **d** Block diagram of the adaptive control technique^95^. **e** CMOS phase-locked loop-driving circuit^59^. **f** Measurement system of the piezoresistive pressure sensor^103^. **g** Closed-loop configuration with the PID technique^104^

The frequency-locking technique seeks to lock the resonant frequency of the RPS to a reference frequency by continuously monitoring and adjusting its operating conditions. The technique consists of several key elements that collaborate to achieve this objective^98,99^, as shown in Fig. 9a and b. The components include frequency detectors that measure the sensor’s resonant frequency, a voltage-controlled oscillator (VCO) generating an adjustable frequency signal, a phase comparator comparing the frequency of the RPS with the reference frequency, and a control circuit that adjusts the operating conditions of the RPS to match the reference frequency. Furthermore, frequency locking has a relatively simple implementation and fast response to frequency variations, which is effective for stabilizing the resonant frequency of the RPS. However, it requires continuous adjustment to compensate for frequency drifts that limit accuracy and stability in the long term, as well as high sensitivity to environmental changes and aging effects.

The phase-locked loop (PLL) technique is a well-known approach for controlling and tracking the frequency of an RPS, as shown in Fig. 9c, d, and e. The operating mechanism of a PLL is based on comparing the phase of the RPS output signal with that of a reference signal and adjusting the operating conditions accordingly^100–104^. In general, a PLL circuit consists of a phase detector that compares the phase of the PRS output with the reference signal, a VCO generating a frequency signal based on the phase difference, a low-pass filter that reduces the signal gain of noise, a high-frequency component, a loop filter shaping the control signal to the VCO based on the filtered output, and a feedback loop connecting the output of the VCO to the sensor to adjust its resonant frequency. A PLL exhibits excellent frequency tracking and stability with improved immunity to environmental changes and can compensate for long-term frequency drift. However, it consumes more power and requires more complex circuitry and design than other techniques.

The adaptive control technique involves continuously adapting the control parameters based on the responses of the RPS and environmental conditions. It comprises five parts: a signal processing unit analyzing the output signal of the RPS and estimating the deviation from the desired frequency, a controller generating the control signal based on the adaptive algorithm, an actuator adjusting the operating conditions of the RPS based on the control signal, and a feedback loop connecting the actuator to the RPS to continuously adapt and stabilize the resonant frequency^105^. Although it has a more complex implementation and algorithm design with high computational requirements and limited effectiveness under extreme operating conditions, the adaptive control technique exhibits enhanced accuracy and stability because it can adapt to changes in sensor characteristics with robust performance under varying conditions.

Digital control technology utilizes digital signal processing (DSP) algorithms to analyze the output frequency of the RPS and generates control signals for frequency adjustment, as shown in Fig. 9e. It consists of five main parts: an analog-to-digital converter (ADC), which converts the analog output of the RPS to a digital signal; a digital signal processor (DSP), which performs signal processing algorithms on the digital signal; a control algorithm, which analyzes the processed data and generates the control signal for frequency adjustment; a digital-to-analog converter (DAC), which converts the digital control signal back to an analog form; and an actuator, which adjusts the operating conditions of the RPS based on the analog control signal. By utilizing digital signal processing techniques, digital control techniques enable high precision, system flexibility, enhanced control capabilities, and simple integration with supporting digital systems. However, these types of implementations increase the complexity of hardware and software, with the potential for additional noise and quantization errors^106^.

The proportional-integral-derivative (PID) control technique is based on proportional, integral, and derivative control actions to adjust the control signal, which includes an error detector, proportional gain component, integral gain component, derivative gain component, and summing amplifier and actuator^74,77,94,107^, as shown in Fig. 9f and g. The proportional gain component multiplies the error signal between the resonant frequency of RPS and desired frequency measured by the error detector to provide proportional control, while the integral gain component integrates the error signal over time to provide integral control, and the derivative gain component calculates the rate of change of the error signal to provide derivative control. Following the combination of the outputs of the proportional, integral, and derivative components by the summing amplifier to generate the control signal, the actuator adjusts the operating conditions of RPS based on the control signal derived from the summing amplifier. Proportional-integral-derivative (PID) control is widely regarded as a used and well-established technique with balanced stability, accuracy, and response speed; however, it requires careful tuning of control parameters to avoid oscillations or overshoots and has limited effectiveness in highly nonlinear or time-varying systems.

In summary, the operating mechanisms of these closed-loop control techniques involve various strategies, such as adjusting the operating conditions, comparing phase differences, adapting control parameters, utilizing digital signal processing, and applying proportional, integral, and derivative actions. Each technique seeks to maintain the resonant frequency of the MEMS resonant pressure sensor at the desired value by continuously monitoring and adjusting the sensor behavior. The choice of closed-loop control technique depends on various factors, including the desired performance, system requirements, and specific application constraints. In MEMS resonant pressure sensor designs, it is crucial to consider factors such as accuracy, stability, power consumption, complexity, and adaptability.

RPSs have been crafted from both quartz and silicon, employing diverse excitation and detection methodologies, as detailed in Table 2. Through various designs, impressive sensitivity, accuracy, and *Q*-values have been attained. However, to date, RPSs have primarily been deployed in pristine environments and for low-pressure detection. Future studies should consider high-pressure range RPSs and refined differential pressure RPSs. Additionally, there is opportunity for enhancement of the frequency response capability of RPSs. The current threshold of 100 Hz constrains the usability of such devices in dynamic pressure measurement scenarios.

**Table 2:** Performance comparisons of typically reported RPS studies

Author	Accuracy %FS	Sensitivity Hz/kPa	*Q*-factor	*TCF* ppm/°C	Detection method	Resonator number
Sun^74,75^	±0.02	29	>10,000	44.4	Capacitance	Not differential
Du^76–78^	±0.02	20	>22,795	271	Capacitance	Not differential
Welham^88^	±0.01	75	>50,000	/	Piezoresistive	Differential
Yan^89^	±0.01	11.89	>16,000	6.5	Piezoresistive	Differential
Han^90^	±0.02	19	>25,000	7.3	Piezoresistive	Not differential

## “Pressure+*x*” integrated chip

In different applications of pressure sensors, parameters such as pressure, temperature, humidity, and vibration often exhibit strong interference. Specifically, in the process of thermal shock, the discrete pressure/temperature chip design has a large temperature-field unevenness, which reduces the sensor accuracy. Therefore, to reduce the influence of other factors on the sensing accuracy, the in situ temperature must be obtained and compensated. Moreover, multiparameter signals, including temperature, pressure, and vibration, are needed for machine health monitoring, and many scenarios have high installation space requirements. Thus, a miniaturized integrated chip must be developed^117–119^. The following subsections introduce the status of silicon-based pressure-related integrated sensors using different integration methods.

### Integrated sensor with discrete chips

**Fig. 10:**
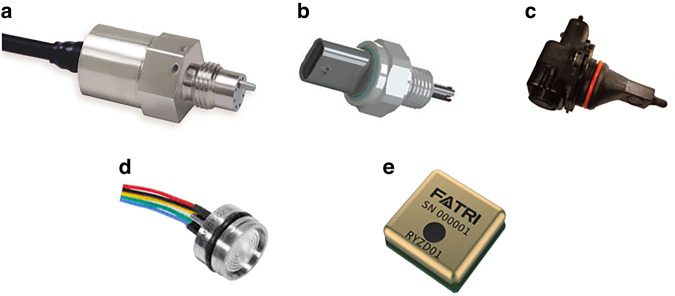
Pressure–temperature sensors developed by different companies. **a** Kulite^120^. **b** Sensata^121^. **c** Amphenol^122^. **d** Microsensor^123^. **e** Fatritech^124^

Discrete packaging refers to the packaging of pressure and temperature sensors via device-level combinations, which are relatively mature in the industry. Kulite^120^ (Fig. 10a) adopted leadless pressure-packaging technology and integrated pressure with temperature for aerospace applications. Sensata^121^ (Fig. 10b) developed a pressure sensor based on RTC resistance and ceramic capacitance for air conditioning systems, and Amphenol^122^ (Fig. 10c) developed an integrated sensor with five parameters, namely, temperature, pressure, and humidity, mainly for air intake. To measure manifold parameters, Microsensor^123^ (Fig. 10d) developed an oil-filled integrated temperature and pressure sensor, and FATRI^124^ (Fig. 10e) developed a temperature, pressure, and humidity composite sensor, mainly for the consumer industrial market. Tian^125^ assembled a platinum temperature chip and SOI pressure chip in an oil-filled cavity. Given that their two chips were arranged as neighbors, the temperature in the cavity changed slightly based on the electrothermal nature of the integrated sensor, which affected the measurement accuracy. The distances between the temperature and pressure sensors were controlled. The test results show that the nonlinearity of the pressure sensor is better than 0.2% FS within 0–75 MPa, and the *TCR* of the Pt sensor is 3850 ppm/°C in the range of −50–175 °C.

### Integrated “pressure + *x*” sensor chip

Multiparameter chips mainly integrate different sensors at the chip level, realizing multisensing miniaturized packaging and in situ measurements. Compared to the discrete package, an integrated chip has a smaller package volume and better in situ measurement and compensation accuracy.

**Fig. 11:**
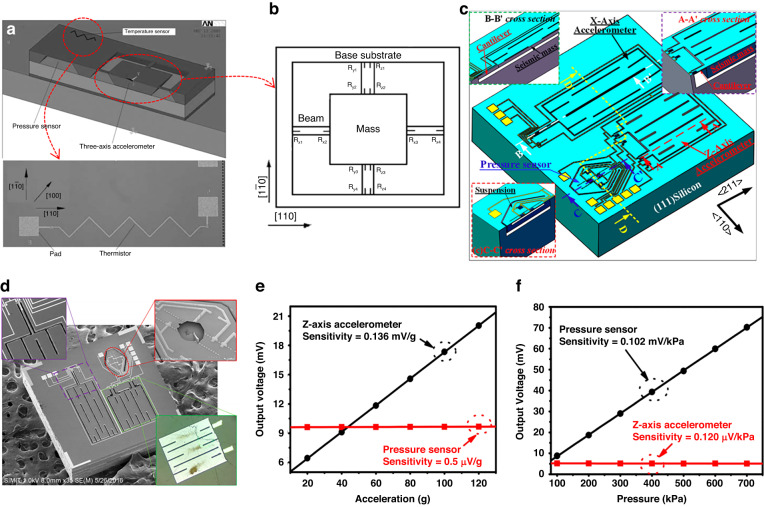
Typical “pressure + *x*” integrated sensor chip. **a** Cross-section of the integrated pressure/temperature/acceleration sensor chip^126^. **b** Arrangement of piezoresistors for the three-axis accelerometer in (a)^126^. **c** Sketch of the on-chip integration of the pressure plus two-axis acceleration composite tire pressure monitoring system (TPMS) sensor^127^. **d** SEM image of the fabricated sensor^127^. **e** X-axis accelerometer output and eliminated cross-sensitivity of the Z-axis accelerometer^127^. **f** Pressure sensor linear output and negligible pressure-induced crosstalk in the Z-axis accelerometer^127^

To satisfy the multiparameter detection requirements of smartphones or TPMS, Zhao et al. ^126^ utilized piezoresistive thermal/stress characteristics and integrated acceleration/pressure/temperature sensors, as shown in Fig. 11a. To reduce the influence of stress on the temperature sensing accuracy, a thermistor is arranged by an unsensitive 45° with a <110> direction in the low-stress area. The pressure sensor adopted a common piezoresistive design, and the three-axis accelerometer used three sets of Wheatstone piezoresistive bridges for data decoupling measurements (shown in Fig. 11b). Finally, the integrated chip size is only 4 mm × 6 mm × 0.9 mm. The sensitivity of the pressure sensor is 0.020 mV/V/kPa, the nonlinearity is 0.4%FS, the sensitivity of the temperature sensor is 0.56 Ω/°C, and the nonlinearity is 0.48%FS. To decrease the chip size and crosstalk between different sensors in integrated chips, Wang et al. ^127,128^ developed pressure/acceleration integrated chips, as shown in Fig. 11c, d. The integration of a pressure/2-axis accelerometer is proposed with an extremely small chip size of 1.9 mm × 1.9 mm and low cross-talk interference (*V*_*pressure*_ = 0.5 μV/g, *V*_*accelerometor*_ = 0.12 μV/kPa, Fig. 11e, f). Dong et al. ^128^ adopted multilayer-assisted bonding technology to ensure sealing of the bonding and signal transition. Moreover, the multilayer design on the bonding surface effectively protected the PN junction from breaking down during the anodic bonding process. A sandwich sealing structure was formed, and the integrated chip size was only 2.5 mm × 2.5 mm × 1.4 mm.

**Fig. 12:**
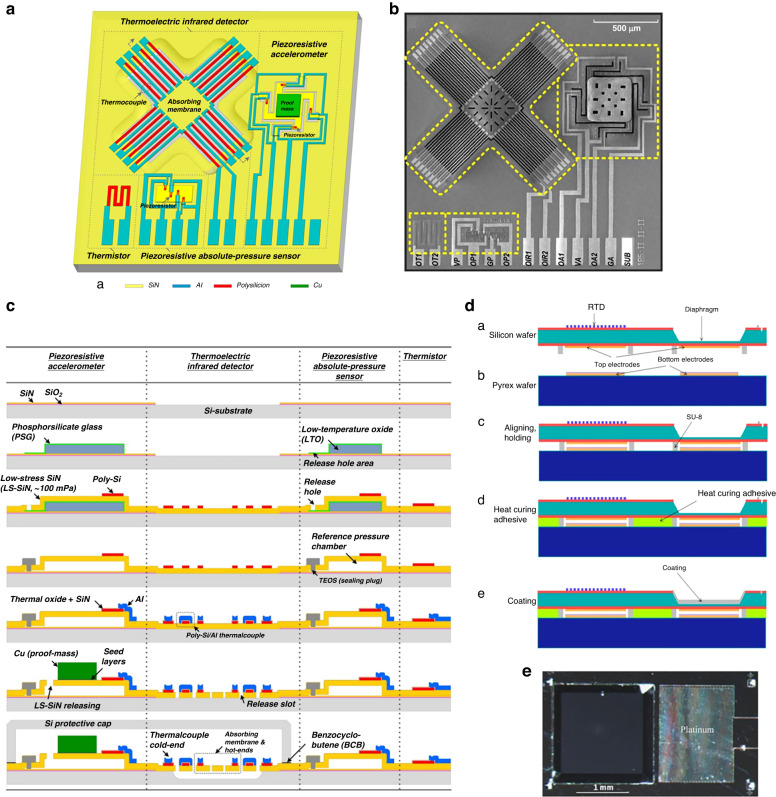
Integrated sensor chip design of “pressure + acceleration + temperature + infrared” (PATIR)^130^. **a** Schematic of the integrated sensor. **b** Photograph showing the prototype PATIR integrated sensor. **c** Developed single-side-integrated process flow for prototype PATIR composite sensor. **d** Fabrication process flow of the capacitance pressure and temperature sensor^132^. **e** Top view of the CPS^132^

In addition to the SCS integrated sensor, Li et al. ^129,130^ used a single-sided micromachining process to prepare acceleration, pressure, temperature, and infrared integrated sensors, as shown in Fig. 12a, b. Specifically, the pressure sensor comprised a low-stress rectangular Si_3_N_4_ diaphragm and polysilicon Wheatstone bridge. The process flow is shown in Fig. 12c. The pressure range is 0-700 kPa, with a sensitivity of 49 mV/MPa/3.3 V and linearity of ±1.2%FS, which is much lower than that of a single-crystal silicon pressure sensor. The temperature sensor is also composed of polysilicon resistance strips, and the sensitivity is 710 ppm/°C and nonlinearity corresponds to ±0.71%FS. The piezoresistive accelerometer sensor exhibits a sensitivity of 66 μV/g (Vin = 3.3 V) and a nonlinearity of ±0.41% FSO. The final sensor size is 2.5 mm × 2.5 mm. Moreover, to form a closed cavity, metallic bonding was used for wafer-level packaging to avoid the influence of external airflow.

Pramanik et al. ^131^ designed a porous silicon-based pressure and temperature integrated sensor within a pressure range of 0–80 kPa and temperature of 25–80 °C. The piezoresistive nanoporous silicon pressure sensor implemented the same working principle as a bulk silicon piezoresistive pressure sensor. However, its sensitivity was three times higher than that of traditional SCS pressure sensors. Furthermore, the sensitivity of the porous silicon heterojunction was improved when compared to that of the traditional PN junction. After sensitivity optimization of the porosity, 55% porosity is selected for the pressure and temperature sensors. The final sensitivity of the pressure sensor is 0.7 mV/V/kPa, and the temperature sensitivity reaches 60 mV/V/°C under reverse bias mode. However, the distance between two sensors must be increased to avoid the cross-coupling effect of the integrated sensor. Moreover, the accuracy of the pressure measurement is limited.

In addition to piezoresistive integrated sensors, Abdolreza et al. ^132^ developed a compensated capacitive pressure and temperature integrated sensor for highly corrosive chemical reactors using adhesive bonding and resistance chemical coating. The fabrication process is shown in Fig. 12d. Specifically, SU8 glue was used for gap control and sealing, and the silicon and Pyrex chips were bonded to form a CPS (Fig. 12e). However, the *CTE* of SU8 is typically high, resulting in a large capacitance temperature coefficient, which affects the accuracy. Platinum was deposited on a Si wafer for temperature detection. To ensure compatibility with the pressure medium, the chip surface was protected using a deposited perylene film. The results show that this design can work in the long term in an environment of 2 MPa@170 °C with a temperature sensing error of ±1.74%FSO and a pressure sensitivity of 0.257 Ff/kPa.

**Fig. 13:**
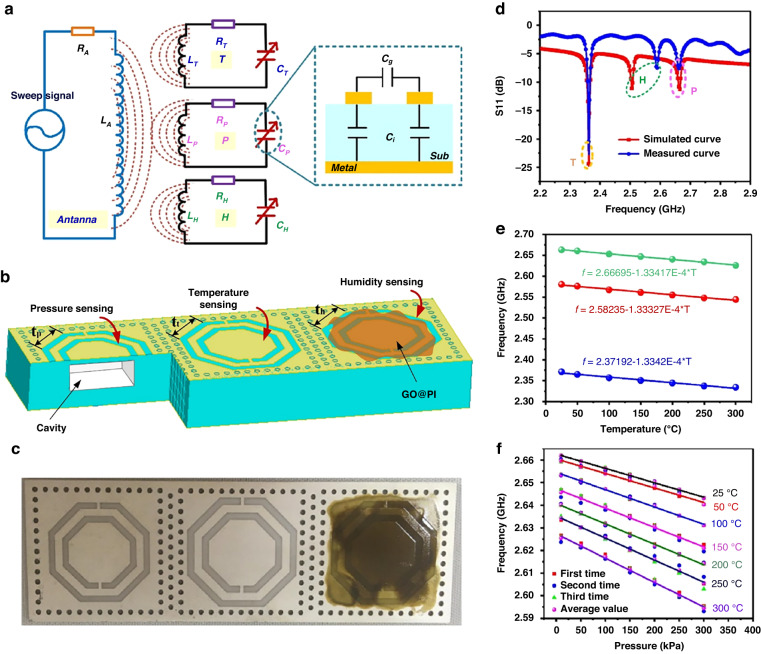
Wireless multiparameter integrated sensor^133^. **a** Schematic and circuit model of the wireless TPH sensor. **b** Structure of the TPH sensor. **c** Photograph of the as-prepared TPH sensor. **d** Simulated and measured frequency distribution of the TPH sensor. **e** Temperature versus frequency curves of the temperature, pressure, and humidity sensors. **f** Pressure versus frequency curve of the pressure sensor at different temperatures within 10–300 kPa

Wireless integrated sensors are typically developed for harsh environments. The wireless integrated temperature–pressure–humidity (TPH) sensor, developed by Tan^133^, is a multiresonance structure with three separate resonant frequencies. The schematic, structure and experimental results are shown in Fig. 13. This design enabled simultaneous measurements of temperature, pressure, and humidity by placing sensitive elements in the corresponding complementary split-ring resonator structures. The TPH sensor can stably work in harsh environments of 25–300 °C, 10–300 kPa, and 20–90% RH.

**Fig. 14:**
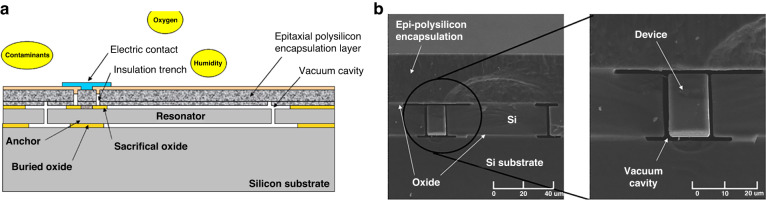
Miniature multiparameter integrated sensor fabricated by the Epi-seal process^135^. **a** Schematic for Epi-seal encapsulated device. **b** Cross section of the Epi-seal sensor

In addition to the integration of temperature, pressure, and acceleration, many researchers have integrated additional parameters for industrial and consumer electronic applications. The integrated sensor proposed by Clifton et al. ^134^ is composed of 10 sensors, including pressure, temperature, acceleration, air speed, and humidity sensors, and it was integrated on a 10 mm × 10 mm chip. During fabrication, only six masks were used, and a common process was developed. The functions and transduction principles of the integrated sensor are listed in Table 3. The piezoresistive strain measurement method was adopted for pressure measurement. To improve the temperature sensor accuracy, in addition to the heavily doped PN junction and Al thermal resistance sensing method, two different currents of the PN diode were used for temperature compensation. The results showed that the pressure sensor exhibited a minimum resolution of 50 Pa within the range of 100 kPa. The aluminum resistance has a final minimum resolution of 0.1 °C. To enhance the sensing accuracy, it is crucial to study the cross-talk among different parameters. Clifton^134–137^ further minimized chip dimensions by adopting a multilayer sensor layout based on the Epi-seal process; a typical design and accompanying image is shown in Fig. 14. The Epi-seal is a hermetic wafer-encapsulation MEMS process, offering a foundation for crafting ultrastable MEMS resonators with high *Q*-factors. This integrated design hosts 10 different sensors within a compact 2 mm × 2 mm package. In this configuration, movable components, such as accelerometers and resonators, are strategically positioned in the central device layer. Moreover, the surface polysilicon encapsulation layer serves as a pressure sensor, with the exterior layer accommodating sensors for elements such as gas and humidity. For high-precision temperature detection, the design employs resonators along with sputtered Al thermal effects, adding a layer of redundancy. This innovative multilayer design significantly reduced the package size of the integrated sensor.

**Table 3:** Sensor function and principles of the integrated sensor proposed by Clifton^134^

#	Sensor functions	Fig. Label	Transduction Principle
1	Temperature	h, i	Resistance detector
		l	Band gap temperature sensor
2	Humidity	c	Dielectric change of polymer
3	Light intensity	m	Photodiode
		b	Doped resistance photodetector
4	Pressure	e	Strain gauge on membrane
5	Airspeed-x	a	Hot wire anemometer
6	Air speed-y	a	Hot wire anemometer
7	Accelerometer-x	f	electrostatic comb fingers
8	Accelerometer-y	g	electrostatic comb fingers
9	Accelerometer-z	d	electrostatic comb fingers
		k	Piezoresistive cantilever
10	Magnetic field	j	Hall effect sensor

In summary, integrated sensor chips have been tailored to accommodate a range of applications, reflecting diverse assembly techniques. With years of development, many challenges associated with integration, especially the complexities of the multilayer stacking process, have been substantially addressed. As evidenced by the performance metrics of various pressure-related integrated sensors in Table 4, the predominant focus of these integrated chips is on the standard pressure range. Notably, there is a lack of studies on integrated sensors customized for high-pressure industrial applications. Although these integrated sensors are mainly employed in multiparameter sensing scenarios, the intricacies of the coupling effect and decoupling mechanism across multiple parameters are not fully understood, highlighting the need for additional research in this domain.

**Table 4:** Research progress of typical pressure-related integrated sensors

Author	Pressure range	Temperature range	Pressure type	Pressure nonlinearity	Integrated sensor No.
Zhao Y^126^	0–200 kPa	−30 °C–150 °C	Piezoresistive	±0.4%FS	P + T + G
Li X^129^	0–450 kPa	−40 °C–100 °C	Piezoresistive	±0.84%FS	P + G
Pramanik^131^	0–80 kPa	25 °C–80 °C	Piezoresistive	/	P + T
Clifton^136^	10–101 kPa	−30 °C–60 °C	Strain effect	/	10 types of sensors
Abdolreza^132^	0–2 MPa	25 °C–175 °C	Capacitance	/	P + T

## Microsized pressure sensor chip

To satisfy the requirements for miniaturized pressure chips in medical and consumer electronics, such as multisensor integration and miniaturized packaging of smartphones and invasive monitoring of intracranial pressure, intravascular pressure, and intrauterine pressure, the width and thickness of pressure chips, in general, should be reduced to <1 mm and <0.2 mm, respectively. With the development of MEMS fabrication technology in recent years, researchers have proposed different schemes to reduce the size of pressure chips. Given that a CPS usually requires a large diaphragm for high sensitivity requirements, miniaturization is difficult to realize. Additionally, optical fiber pressure sensors cannot be used in many applications because of their limited accuracy and complex modulation. Thus, piezoresistive chips are mostly used in miniaturized applications.

**Fig. 15:**
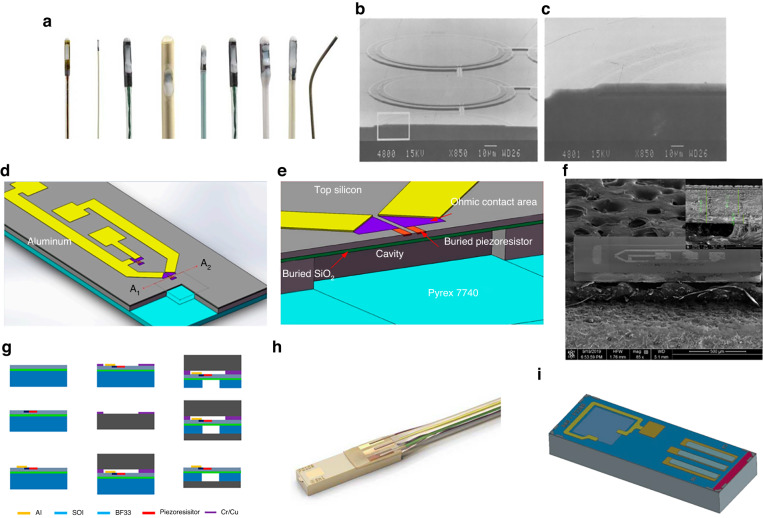
Multilayer bonding of ultrasmall-sized pressure sensors. **a** Ultrasmall-sized pressure sensor developed by Millar (https://millar.com/content/documents/Knowledge_Center/Document_Library/OEM_Resources/Millar-MEMS-Pressure-Sensors_V5_1.pdf). **b** SEM of the cross-sectional view of the capacitive pressure sensor^139^. **c** Magnified view of the diaphragm in Fig. 15b^139^. **d** Structure of an extremely thin absolute pressure sensor^140^. **e** Cross-sectional view of diaphragm in Fig. 15e.^140^. **f** SEM image of the sensor die after separating structures^140^. **g** Fabrication of an extremely thin sensor^140^. **h** Ultraminiature catheter tip pressure sensor developed by SMI (https://www.te.com/usa-en/product-SMI-1B-48-180-BAUU.html). **i** Catheter tip pressure sensor developed by Amphenol (https://www.amphenol-sensors.com/en/novasensor/pressure-sensor-die/3426-p330b)

For the miniaturization of pressure chips, Millar Company^138^ (https://millar.com/content/documents/Knowledge_Center/Document_Library/OEM_Resources/Millar-MEMS-Pressure-Sensors_V5_1.pdf) developed an ultrasmall pressure sensor based on an SOI wafer with a range of 300 mmHg, as shown in Fig. 15a. This device can satisfy the pressure chip requirements of a 1-french (D = 0.35 mm) catheter. The miniaturized chip comprises a top film thickness of only 2.5 μm, and a DRIE process is used to dice the chip and overcome chip cracking issue introduced by slice griding^138^. The final size of the chip is only 650 μm × 230 μm × 150 μm with a high accuracy in the pressure range of 300 mmHg. However, this sensor is a gauge pressure sensor, which is not suitable for absolute pressure sensing. Moreover, to decrease the overall chip size, an integrated capacitive pressure sensor is also fabricated via an IC process with COMS readout electronics^139^. Furthermore, the membrane diameter is only 50 μm, and the linearity is better than that of 1% FS, as shown in Fig. 15b, c.

In addition to the thin pressure film, the ultraminiature chip faces significant challenges in overall chip thickness control. The thickness of an ultraminiature pressure chip should typically be <100 μm for applications in intracranial pressure monitoring. Given the high brittleness risk of ultrathin wafers, their transfer should be addressed first during the preparation process. Song et al. ^140^ proposed a half-bridge miniature ultrathin piezoresistive pressure sensor (Fig. 15d–f) using temporary Cu–Cu hot-press bonding technology. In this sensor, a transitional bonding glass layer functions as a temporary holding and transferring ultrathin crystal plate during fabrication, as shown in Fig. 15g. The final pressure sensor chip size is 1600 μm × 650 μm × 104 μm, the pressure film size is 100 μm × 100 μm × 2 μm, the thickness of the silicon layer is only 77 μm, and the thickness of BF33 glass is only 27 μm. The miniature pressure sensor was installed in a medical catheter that was used to measure blood pressure.

A miniature pressure diaphragm is typically only a few micrometers in size. Thus, it is too fragile, and the dicing and packaging processes easily cause the diaphragm to crack. Therefore, a group from Norway University^141^ proposed a protected pressure diaphragm dicing process based on a protective film tape for implantable MEMS pressure sensors. In their dicing process, the diaphragm cracking problem is avoided. The thickness of the pressure diaphragm was only 1 m, and the chip size was 700 m × 700 m. Moreover, flip-chip packaging for the leadless connection of the chip was completed.

In terms of chip miniaturization, the overall thickness of the absolute pressure chip is extraordinarily thin (usually <100 μm). Thus, fabrication is extraordinarily difficult. Henry et al. ^140,142^ of SMI developed an implantable miniature absolute pressure sensor via anodic bonding (Fig. 15h). Mechanical grinding combined with the HF wet etching process is introduced in the wafer thinning process, which largely eliminates the grinding stress, finally realizing a total chip thickness of 74 μm. Vacuum and miniaturization packages were realized by doping lead technology. The chip surface size was 240 × 900 μm. The accuracy is better than ±0.3%FS, and the developed intracranial pressure chip has been successfully commercialized (https://www.amphenol-sensors.com/en/novasensor/pressure-sensor-die/3426-p330b). Furthermore, Amphenol proposed another miniaturization chip with a thickness of 120 μm and width of 330 μm, as shown in Fig. 15i.

**Fig. 16:**
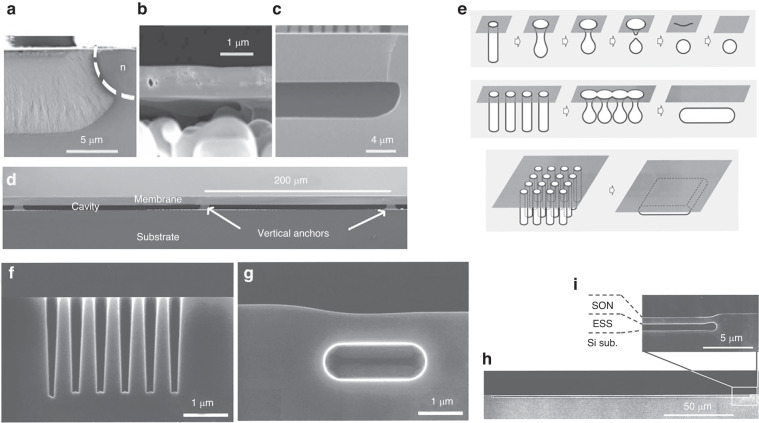
Preparation of suspended pressure diaphragms based on a single wafer. **a** Porous etching of the APSM process^144^. **b** Rearrangement of porous silicon^144^. **c** Epitaxial growth of membranes^144^. **d** SEM of a membrane wafer processed with the APSM process^144^. **e** SoN PC pressure sensor process flow^151^. **f** Initial shape of rectangular trenches with the SoN process^151^. **g** Pipe-shaped empty space due to hydrogen annealing^151^. **h** SEM image of the plate-shaped ESS with an area of 180 μm × 1500 μm and a thickness of 0.7 μm based on the SoN process^151^, **i** Enlarged view of the diaphragm based on SoN process

To obtain a sealed cavity in the pressure sensor chip, a couple of wafer bonds are usually adopted, such as Si-Si and Si-glass, which leads to high cost and chip size. To address this issue, some researchers have attempted to fabricate absolute pressure sensors on one side of a wafer. For example, Bosch developed advanced porous silicon membrane process (APSM) technology^143–149^ to achieve a miniaturized chip. The processing technique is shown in Fig. 16a-c. Porous Si is obtained via electrolysis. Subsequently, a vacuum-sealing cavity is realized due to the molecular diffusion effect of the pressure diaphragm via high-temperature annealing. This method was used to mass-produce capacitance and piezoresistive pressure sensors with a sensor chip size <0.6 mm × 0.6 mm^143–149^. The typical membrane produced by the APSM is shown in Fig. 16d.

As shown above, the polysilicon process based on electrolysis in APSM is complicated. Another single-side cavity process, the silicon on nothing (SoN) process, was developed by Toshiba and realized single-side sealed cavity fabrication^150–156^ In the process, microscale holes are initially etched. This step is followed by annealing under a high-temperature hydrogen atmosphere. The process flow is shown in Fig. 16e, and SEM micrographs of key steps are shown in Fig. 16f, g. In the most important step of annealing sealing, temperature- and time-dependent transformations were observed. Given the shrinkage creep effect of silicon material at high temperatures (normally >1000 °C), a relatively large flat pressure film is easily achieved after annealing for >30 min. This method can effectively control the final thickness of the SoN by controlling the shape and layout of the etched holes. The pressure diaphragm developed through this process is usually thin due to the creeping limit, and it is suitable for sensors with a small pressure range, as shown in Fig. 16h and i. Given that the process difficulty of SoN is so high, it has not been widely popularized.

**Fig. 17:**
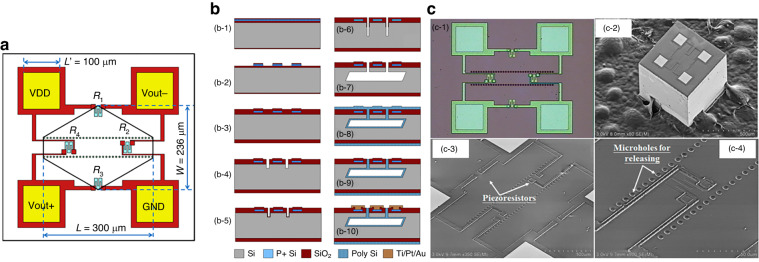
Pressure sensor based on MIS single-sided processing^158^. **a** Schematic of the single-sided processed pressure sensor. **b** Process flow of the MIS pressure sensors. **c** Images showing the fabricated pressure sensor. (c-1) Optical view of the sensor chip, (c-2) SEM view of the pressure sensor chip, (c-3) magnified view of the piezoresistors, and (c-4) magnified view of the microscale holes that were later sealed

To develop a “single side process” absolute pressure sensor chip, Li et al. developed the microhole interaction and sealing (MIS) process based on the crystal direction selection characteristics of a silicon crystal surface (111) via wet etching^157–159^. Compared with the traditional “double side process” MEMS pressure sensor, MIS does not require double-sided alignment and wafer bonding, and it can effectively reduce the chip size and temperature disturbance effect on diaphragm stress. The 750-kPa sensor chip produced by MIS is only 0.6 mm × 0.6 mm^157^, as shown in Fig. 17a, and the nonlinearity is ±0.09%FS. In the MIS process, different materials and high-temperature bonding processes are not involved. Thus, this approach effectively reduces the residual stress. Finally, the *TCO* is only −0.032%/°C•FS^157^. The process flow is shown in Fig. 17b, including the following: (b-1) masking layer formed via thermal oxidation; (b-2) ion implantation and annealing process; (b-3) fabrication of masking layers of silicon nitride and silicon oxide; (b-4) two rows of microscale holes along the <211> orientation are opened via an RIE and a DRIE process sequentially to define the thickness of the pressure-sensing diaphragm; (b-5) a 0.4-µm thick TEOS layer is deposited via an LPCVD process to cover the hole surface; (b-6) microscale holes in the (111) handle layer are vertically etched again via RIE to remove the TEOS layer at the bottom surface, and DRIE is used to deepen the holes; (b-7) SOI wafer is dipped into the anisotropic etchant of 25 wt. % TMAH at 85 °C for ~2 h to form the pressure-sensing diaphragm and cavity via lateral underetching; (b-8) a 4.0-µm thick low-stress polysilicon is deposited via an LPCVD process to seal the microscale holes; (b-9) polysilicon in the front side of the SOI wafer is removed via maskless DRIE; and (b-10) a Ti/Pt/Au film is sputtered and patterned, and chip fabrication is completed, with images shown in Fig. 17c.

**Fig. 18:**
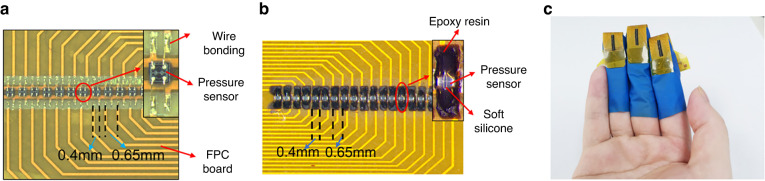
Pressure sensor chip fabricated by the scar-free MIS process^186^. **a** Chips were attached to the FPC board. **b** The chips were coated with silicone and epoxy resin. **c** Pulse sensor arrays were designed to be sufficiently small to be worn on the fingers

In MIS, holes are opened on the pressure diaphragm and appear as scars. Ding et al. ^159^ developed a new scar-free MIS pressure sensor to reduce the influence of hole etching and filling on the residual stress of the pressure diaphragm, thereby significantly improving the product yield and reducing the influence of accuracy. The sealing hole is moved from the deflection diaphragm area to the nondeformation area of the diaphragm based on the new etching hole layout design. Thus, the film remains flat and smooth, which determines the diaphragm stress state. Finally, the miniaturization of the pressure sensor chip is further improved, and the chip size is only 0.4 mm × 0.4 mm, with the scar away from the diaphragm. Given a total silicon design, the nonlinearity is only 0.1%FS, *TCS* is only 0.2%FS/°C, and *TCO* is only −0.064%FS/°C, which is much better than bonded chips. A scar-free sensor chip can then be applied to many microsized package applications at extremely low cost, such as pulse sensing with array packages, as shown in Fig. 18.

**Fig. 19:**
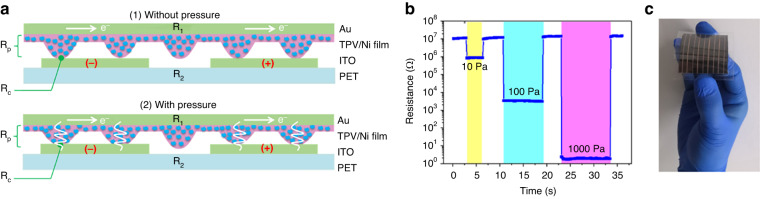
Flexible ultrasensitive thin-film pressure sensors^161^. **a** Working mechanism of the pressure sensor: unloaded (upper) and under pressure (lower) (side view). **b** Resistance response to loading/unloading cycles at various pressures. **c** Photograph of the flexible sensor array for precise measurement of pressure distribution

In addition to the commonly used silicon MEMS-miniaturized pressure sensors, flexible pressure sensors are also becoming increasingly miniaturized due to their favorable biocompatibility and surface adhesive properties. Willyan et al. ^160^ proposed an implantable strain pressure sensor based on the composite structure of polyimide film and SU8 adhesive. The overall thickness of the sensor is only 104 μm, and the film thickness of the pressure film is 3 μm. Given that an extra package is not needed, the size of the sensor is much smaller than that of MEMS products, such as Millar. Mikrotip pressure catheters are adopted to realize ultrathin flexible chip fabrication via wafer-level, silicon transfer, and metal release processes, realizing high-precision width control of the organic diaphragm. Based on this method, a new method was proposed for an ultrathin organic micropressure sensor; however, Pt was selected as the strained material, which hardly realizes the decoupling of temperature and pressure signals, resulting in low measurement accuracy. However, the long-term stability and compatibility of organic diaphragms still require further improvement. In addition to flexible diaphragm-based pressure sensors, flexible contact-based pressure sensors are also commonly used for extremely sensitive pressure sensors, and their typical mechanism is shown in Fig. 19a. *R*_*p*_ is related to pressure with an isotropic piezoresistive effect. The resistance response of the resolution, proposed by Tian^161^, reaches 10 Pa, as shown in Fig. 19b, and the sensor arrays are shown in Fig. 19c.

**Fig. 20:**
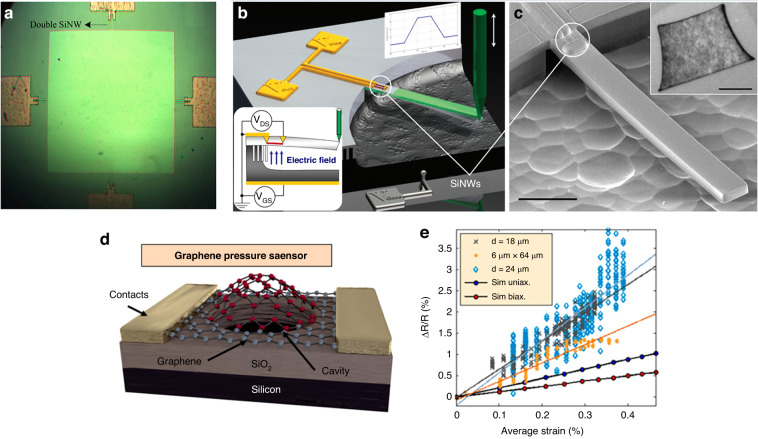
Silicon nanowire sensor and miniaturized graphene pressure sensor. **a** Photograph of the NW pressure sensor^163^. **b** Schematic of the test setup, showing 5-µm long NWs (red) embedded next to the anchor of the SiO_2_ cantilever^165^. **c** SEM image of released cantilever with embedded NWs^165^. **d** Schematic of the graphene pressure sensor common design. **e** Percentage change in resistance of graphene membrane area for three devices with different membrane areas^166^

The emergence of new sensitive materials, such as silicon nanowires and graphene, has paved the way for innovative approaches to pressure chip miniaturization. The length of the silicon nanowires used in pressure sensors is only a few micrometers, and the width is only hundreds of nanometers. Furthermore, the pressure diaphragm width can be controlled within 100 μm^162–165^, as shown in Fig. 20a–c. Moreover, given that silicon nanowires are mainly fabricated by electron beam lithography, the mass manufacturing process is highlighted in subsequent research to reduce the cost of sensor preparation. The piezoresistive graphene pressure sensor mainly uses the strain effect of graphene for resistance detection. The common structure is shown in Fig. 20d. Given that the graphene film is composed of a single layer or multiple layers of carbon atoms, its thickness is only subnanometer or nanometer, and the width size of the pressure film is only in the range of 2–5 μm. This design thus allows for chip miniaturization. However, during the fabrication of the graphene pressure diaphragm, significant folds and film stress can arise, leading to substantial nonlinearity^166^. Moreover, its sensitivity was found to be low, exhibiting a dimensional strain effect on the resistance, as depicted in Fig. 20e. Thus, upcoming research should prioritize enhancing the linearity and sensitivity of these sensors.

## Leadless package pressure sensor

**Fig. 21:**
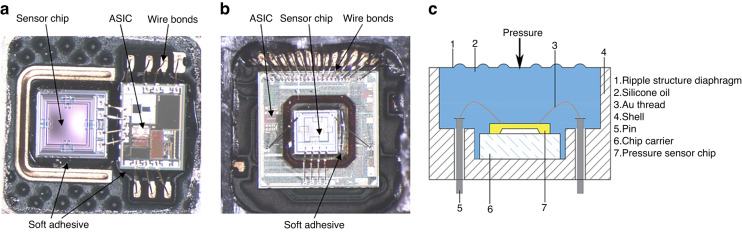
Conventional industrial pressure sensor package form^167^. **a** BMP180 pressure sensor developed by BOSCH. **b** LPS331AP pressure sensor developed by ST Microelectronics. **c** Schematic of the package with silicon oil to protect the leads

Packaging is a crucial step for MEMS pressure sensors. Not only does the package protect the device from external influences, but it also ensures a pristine and stable environment for sensor operation. Contaminants can significantly degrade the performance of the sensor. MEMS pressure sensor chips are often encapsulated using wire bonding paired with an oil-filled cavity^167^, as illustrated in Fig. 21. Direct wire-bonding packaging does not offer protection for either the chips or the gold wires. Due to its affordability and ability to protect from corrosion, direct bonding is predominantly employed in consumer electronics, exemplified by devices such as BOSCH BMP180 (Fig. 21a) and STMicroelectronics’ LPS331 (Fig. 21b). For oil-filled pressure sensors, silicon oil, known for its excellent media compatibility, serves as the protective medium for the pressure-sensitive chip and bonding wire^168^ (Fig. 21c). However, such a larger package not only requires significant mounting space but also comes at a higher cost. Considering that the medium generally lacks resistance to high temperatures, it is unsuitable for certain extreme high-temperature (>250 °C) settings. Moreover, the frequency response of oil-filled packages diminishes, as a lengthy pressure conduction pathway is established from the metal diaphragm to the chip’s surface (Table 5).

**Table 5:** Leadless package pressure sensor characteristics of reported articles

	Pressure range	Temperature range	Sensor chip	Nonlinearity	Leadless type
Wang^170^	5.0 MPa	150 °C	1.5 mm × 1.5 mm	0.076%FS	TSV
Tian^179^	0.7 MPa	450 °C	4.8 mm × 4.8 mm	0.18%FS	Sintering
Dong^172^	1.6 MPa	250 °C	3 mm × 3 mm	0.39%FS	TGV
Xie^183^	3.5 MPa	200 °C	3 mm × 3 mm	0.1%FS	Sintering
Tian^168^	4.5 MPa	300 °C	3 mm × 3 mm	0.4%FS	Sintering

Two main solutions to address these challenges are flush packaging^169^ and leadless packaging^170–172^. Flush packaging is primarily employed for high-dynamic pressure measurements. However, its chip size tends to be larger, as the chip is typically wire-bonded vertically to the circuit and then sealed with a stainless-steel base. For more compact designs with stringent reliability criteria, leadless packaging omits the need for wire bonding, enabling the chip to interface directly with the PCB or base. This approach facilitates electrical signal transmission through a vertical conductive path, establishing vertical interconnections between chips and bases. Thus, it offers superior strength, miniaturization, and a high-density signal connection. Leadless packages also offer benefits such as a reduced interconnection pathway, minimized electromagnetic interference, robust sealing, and excellent long-term stability. Depending on the chip connection approach, leadless pressure sensors are mainly categorized into flip-chip (FC) packages and integrated sintering packages. Subsequent sections will investigate the designs and performance of these distinct packaging methods.

### Pressure sensor based on the FC package

The FC packaging method is prevalent in consumer electronics, including CPUs, memory chips, and sensors. Based on the chip connection style, packaging methods can be categorized into through-silicon via (TSV) and through-glass via (TGV).

**Fig. 22:**
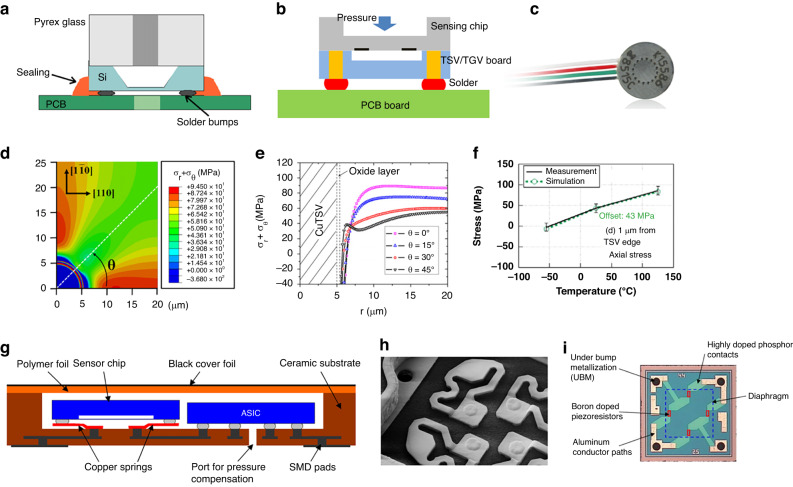
FC-based pressure sensor. **a** Cross-sectional view of the FC gauge pressure sensor chip. **b** Cross-sectional view of the FC absolute pressure sensor chip. **c** Endvoco 8515 C pressure sensor-based FC package^173^. **d** Contour of the thermal stress near the Si wafer surface,^175^.**e** Directional dependence of the stress distribution in (d) (*ΔT* = –270 °C)^175^. **f** Comparison of thermal stress using FEM simulation and measurement data by Raman spectroscopy^187^. **g** Sensor chip mounted on copper springs to reduce mechanical stress on the sensor chip^167^. **h** Copper springs in package cavity^167^. **i** Top view of the FC chip^167^

Unlike conventional TSV IC chips, pressure sensors are notably more sensitive to the stress state. Given that the material of the TSV layer aligns with that of the pressure-sensing layer, thermal stress over a broad temperature range is lower in TSV than in TGV sensors. In leadless packaging, the sensor chip is predominantly soldered onto the front side of the PCB, considerably reducing the sensor’s overall packaging size. The typical packaging methods for gauge and absolute FC pressure sensors are illustrated in Fig. 22a and b. For example, the TSV pressure sensor crafted by Wang et al. ^170^ spans dimensions of only 1.5 mm × 1.5 mm. This sensor has a pressure range of 100 kPa, a chip thickness of 400 μm, and an overall sensor thickness ranging from 0.9–1 mm. Both Endevco^173^ (refer to Fig. 22c) and BCM^174^ have also developed ultrathin TSV pressure sensors for high dynamic applications with a package thickness of 0.76 mm.

As with FC sensors, a TSV sensor is usually filled with copper or other metals (e.g., *CTE*_*Cu*_ = 17.5 ppm/°C, which is much higher than the *CTE* of silicon [2.5 ppm/°C]). Consequently, in a wide temperature range, substantial thermal stress is generated, and the accuracy is worsened. The impact of TSV-induced stress on the thermal performance of pressure sensors has been a focal point for many researchers by simulation and experiment. Studies have revealed that temperature fluctuations (*ΔT* = −270 °C) induce considerable stress (>90 MPa) around the TSV hole, as depicted in Fig. 22d and e^175^. This can be corroborated using polarized Raman spectroscopy with high precision, as demonstrated in Fig. 22f. To mitigate the thermal stress arising from the TSV filler, Yvonne et al. ^176^ introduced a TSV design based on Si pillars. This Si-TSV primarily employs a deep-silicon etching isolation trench process to ensure electrical insulation. The surface was sealed using SiO_2_ to close off the isolation trench and to attain surface planarization. This approach dramatically reduces the thermal stress. Moreover, this process will also introduce residual stress and process complexity, which will still need further research. Additionally, the interconnection structure of the Redistribution Layer (RDL) used in the Si-TSV is metal, so it may still result in large stress and needs later research.

Currently, sensors based on TSV technology are widely used in the fields of piezoresistive pressure^174,176^, CPS^177^, and acoustic sensors^178^. However, the TSV package sensor has two major drawbacks: (1) TSV fabrication is complicated, including silicon etching, oxidation, and electroplating filling, introducing high cost and a long process, and (2) a bonding-aided layer is commonly needed in silicon bonding. Borosilicate glass has no free-moving charges, excellent dielectric properties, favorable airtightness, favorable stability, and low cost. Many research institutions have applied TGV to develop pressure sensor chips; however, TGV exhibits low surface requirements (such as roughness and total thickness variation) for wafer bonding, which introduces less fabrication difficulty.

Dong et al. ^172^ proposed an FC packaging pressure sensor based on the TGV process. An FC SOI high-temperature pressure-sensor chip was designed and fabricated. In this design, chip-level Au–Au bonding is selected because of its excellent electrical conductivity and bonding performance, as opposed to the common soldering method. This leads to the realization of high-temperature connection resistance between the chip and ceramic PCB. The sensitivity of the sensor was 8.69 mV/100 kPa, and its basic error was <0.39%FS; however, this process hardly achieved mass production because of the difficulty in chip-level bonding. Similarly, Tian^179,180^ developed another high-temperature leadless packaged silicon pressure sensor using a TGV and optimized the thermal stress under extreme temperature differences. The test results show that the nonlinearity is 0.18%FS in the temperature range of 20–450 °C. The thermal zero-point drift is only 5.71 mV, and the full-scale output temperature drift is only 8.45 mV. However, the chip size is ~4.8 mm × 4.8 mm, and the diameter of the packaged sensor is >10 mm.

In an FC packaged sensor, given the *CTE* mismatch of the material between the base and chip, a large packaging stress often occurs. Reducing the packaging stress is key to improving accuracy. To reduce the stress due to solder joints, Waber et al. ^167^ developed a copper spring connection soldering structure (Fig. 22g). The package stress was significantly reduced via a flexible spring connection (the MEMS spring is shown in Fig. 22h, and the FC chip is shown in Fig. 22i). Hysteresis of the sensor accordingly decreases from 140 to 20 Pa. However, this method is only suitable for static pressure measurement and is not suitable for high dynamic pressure measurement or strong vibration measurement due to its low connection stiffness.

In general, the FC pressure sensor easily achieves miniaturized packaging and a high-frequency response because the chip is primarily packaged with a Pb–Sn solder and mounted in the front face. However, the solder softening temperature is usually low, and its ultimate strength and stiffness limit its application in FC design at high temperatures. In the FC structure, the underfill effectively protects the solder joints from the particles. However, given the *CTE* mismatch between the underfill, silicon chip and printed circuit board (PCB), nanoscale gaps inevitably exist after a long period of pressure and temperature cycling. Thus, this FC pressure sensor hardly meets the requirements of conductive or corrosive media. Thus, chip-size protection methods should be investigated.

### Integrated sintered package pressure sensor

**Fig. 23:**
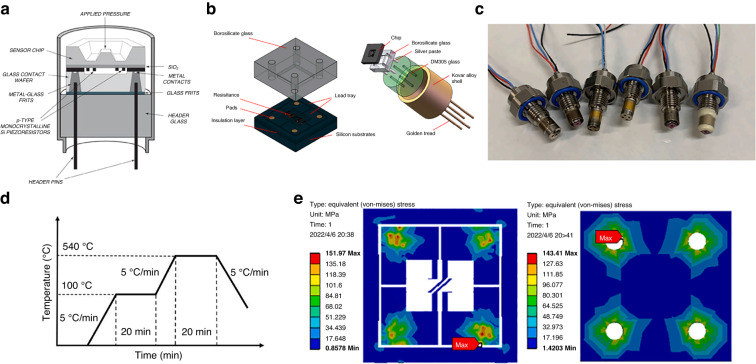
Leadless package pressure sensor via the sintering method. **a** Kulite leadless package structure^181^. **b** Package design proposed by Zhong^182^. **c** SOI high-temperature pressure sensors in leadless packages,^182^. **d** Sintering profiles of silver and glass paste^182^. **e** Maximum stress cloud at 200 °C, 3.5 MPa, and random vibration^182^

Pressure sensors based on glass sintered packaging with smaller packages and improved performance have been developed for decades to satisfy the requirements of medium compatibility and solve the organic package material creeping issue. Leadless package sensors based on glass sintering exhibit better temperature adaptability and sealing characteristics because the sealing glass completely isolates the chip electrode and pressure medium. For example, researchers have developed various pressure sensors based on glass-sintering technology for high-temperature applications^181,182^ (Fig. 23a, b). Additionally, the sintering package can meet the operational demands at temperatures exceeding 400 °C.

To enhance accuracy across a broad temperature spectrum, it is essential to first minimize the stress in the sensor’s sintering leadless packaging. Due to the CTE discrepancies between the chip and packaging material, significant thermal stress is produced, negatively impacting temperature stability. To mitigate this sintering stress, Tian et al. delved into the primary contributors to the leadless packaging stress using the “Taguchi” method^168^. Factors examined included the thickness of the packaging glass layer, diameter of the conductive hole, thickness of the glass base, and metal shell thickness. The findings indicate that the diameter of the silver paste hole plays a pivotal role in packaging stress. The sensor chip, with dimensions of 3 mm × 3 mm × 0.5 mm, is leadless packaged as depicted in Fig. 23c. Following optimization, the packaging stress is reduced by 16.65%. Concurrently, this pressure sensor exhibited a sensitivity of 30.82 mV/MPa, with a nonlinearity below 0.40% FS.

During the glass sintering procedure, the densification of the glass layer and conductive silver has a significant impact on the sensor’s vibrational and corrosion resistance. To enhance the reliability of leadless packaged pressure sensors within this sintering phase, Xie et al. ^183^ utilized nanopaste to fill glass holes. They delved into the sintering mechanics of both the silver and glass pastes (see Fig. 23d) and analyzed the critical parameters influencing connection strength, vibration resilience, and glass porosity. The final integrated sintering procedure, which amalgamates the nanosilver paste with the glass paste, was derived from comprehensive sintering and vibrational stress simulations (depicted in Fig. 23e). This approach aided in optimizing the composition and procedure for both the glass layer and silver electrode.

In addition to SOI pressure chips, Masheeb developed a leadless packaging technology for 6H-SiC pressure sensors^184^ to improve the temperature range. Similarly, the electrical interconnection between the sensor chip and base was composed of a metal–glass mixture. The glass frit for connection and metallic glass frit for electrical contacts are fired simultaneously at 650 °C.

In summary, pressure sensors based on FC leadless packaging have been used in industrial and consumer electronics and have realized miniaturized packaging and highly dynamic measurements; however, their packaging stress should be reduced further. Researchers have developed pressure sensors based on an integrated sintering process to achieve high-temperature packaging and media compatibility. However, pressure sensors based on the sintering method usually require a large chip size because a sufficient lead distance in the glass base is needed during assembly. Thus, the smallest reported sensor chip currently measures 3 mm × 3 mm. However, the packaging stress is still considerably high, necessitating further reduction to enhance temperature stability.

## Discussion

To elaborate on these development trends of MEMS pressure sensors in detail, several typical pressure sensors developed in recent decades are described and analyzed in this review. Although MEMS pressure sensors with common specifications have been successfully commercialized, several technical difficulties still need to be addressed.

### MDPS

As discussed, piezoresistive MDPS design challenges encompass achieving extremely high sensitivity, a swift frequency response, and minimal chip dimensions. Additionally, a sensitivity-frequency coupling tradeoff exists. As various research has suggested, the key to amplifying sensitivity is to enhance the stress concentration effect. Toward this goal, distinct structures, including combinations of “beam,” “island,” and “diaphragm,” have been proposed. Of these, the “diaphragm + island + peninsula” configuration offers the maximum sensor sensitivity. When considering chip dimensions, the presence of a large thin diaphragm hampers the potential for miniaturization, which is crucial for effective cost management.

Compared with standard pressure-range sensors, the primary challenges in MDPS fabrication revolve around the production of ultrathin pressure membranes and precise control of shallow PN junctions. Given that the membrane thickness for the majority of MDPS is below 10 µm, the thickness of the passivation layer, metal layer, and depth of the PN junction on the membrane can significantly influence the stress state, thereby directly impacting accuracy and sensitivity. Thus, to enhance accuracy, future studies should aim to stabilize and regulate the film stress.

In addition to exploring piezoresistive MDPS devices, there is potential to further explore capacitive and resonant MDPS. Currently, in industrial settings, there is a notable lack of reports on capacitive or resonant MDPSs. The majority of metrology-grade gauge pressure sensors at the kPa level remain as RPS, while metrology-grade vacuum pressure sensors at this level are predominantly achieved through CPS.

For one step further, a smaller pressure range (<100 Pa) is urgently needed in infant ventilators. Thus, the sensitivity and accuracy of the sensor must be further improved. The dynamic performance of the MDPS must be further improved in various fields, including shockwave measurements and wind testing.

### RPS

An RPS is often developed for high-precision pressure measurements with excellent temperature stability and pressure resolution, which are very important for monitoring the full temperature range. Different RPS materials have been proposed, and quartz and silicon RPSs are most widely used due to their high accuracy.

Quartz and silicon RPSs with different excitation and detection methods have been proposed by different researchers. Quartz RPSs have been used in ultrahigh-pressure range applications, such as oil drilling pressure measurements, and in small pressure ranges, such as flight altitude sensing. Moreover, quartz resonator fabrication, especially the etching process, is more difficult than silicon fabrication, and researchers should more closely consider the capability to fabricate and assemble these systems. Moreover, the closed-loop control circuit for quartz is more mature than that of silicon RPS, promoting wide application of the former.

For silicon RPS, the reported sensor accuracy is better than 0.01% FS, and the chip size has been greatly reduced. Most research has been focused on TCF control because the TCE of silicon is as high as −60 ppm/°C, which leads to a TCF of −32 ppm/°C. The differential method using dual resonators is prevalent, allowing the TCF to be reduced to below 10 ppm/°C. Another method, which leverages thermal stress TCF effects and TCE counteraction, has successfully lowered the TCF to 7.2 ppm/°C. To enhance the sensing accuracy, there are potential avenues for further reducing the TCF, such as employing composite material compensation and modulating the elastic modulus via heavy doping. Nevertheless, future research should investigate swift temperature compensation techniques, which are vital for complex flow fields and environments with rapidly fluctuating temperatures. Temperature sensors should be integrated closely with a pressure sensor chip, ideally adjacent to the resonator sensing beam, to negate the effects of uneven temperature distributions.

Due to the limitations of the compensation circuit, the response time of the existing RPS sensor is usually >100 ms. Thus, it is difficult to measure high dynamic pressures. Therefore, the dynamic compensation circuit must be improved to realize fast frequency locking and control. Additionally, most RPSs can only survive in clean environments and not in harsh applications with simple packages. In the future, emphasis should be placed on novel packaging methods used for various fluid media.

### Integrated “Pressure+*x*” sensor

Based on varying application needs, researchers have developed sensors that integrate various combinations of “pressure+*x*” onto a single chip using compatible fabrication processes. These integrated sensors fulfill the demand for simultaneous multiparameter detection in automotive and consumer electronics.

To minimize measurement discrepancies arising from the discrete-device-packaging method, multiple parameters are sensed simultaneously, and the precision is enhanced in compensation. Moreover, the evolution of integrated fabrication has moved from 2D “in-plane” integration to multilayer 3D processes, including methods such as the “EPI” process. This progression paves the way for further miniaturization of sensor chips, making them even more suitable for space-constrained packaging applications.

Furthermore, several challenges remain to be addressed for the “pressure + *x*” sensor chip. These encompass understanding the interaction mechanism of multiple parameters, refining multiparameter compatible fabrication, and developing circuit interfaces. Integrated sensors are typically deployed in multifaceted environments characterized by variables such as temperature, vibration, and humidity. Initially, the interplay between different sensor units was studied and isolated through thoughtful packaging design. From a fabrication standpoint, the need for numerous masks or steps for the integrated sensor introduces substantial quality control challenges. In the future, designs that prioritize process compatibility should be emphasized.

In future research, both the packaging method and compensation circuit must be examined in tandem. Such a dual focus is paramount for optimizing sensor performance, especially in extreme conditions.

### Microsized pressure chip

Microsized pressure sensors are predominantly utilized in medical implantable pressure monitoring, consumer electronics for altitude tracking, and compact packaging for pulsating pressure measurements, among other applications. In this context, the term microsized pressure sensor specifically denotes the absolute pressure sensor. Fabricating these devices is more challenging than fabricating gauge pressure sensors due to their requirement of a sealed cavity, which is typically achieved through a pair of bonded wafers. The combined thickness of these dual-layer wafers tightly constrains the in-plane and out-plane dimensions of the sensor chip.

Various structures and fabrication methods have been introduced to address these challenges. Notable efforts include the APSM process proposed by BOSCH and the SoN process by Toshiba. These methods have substantially reduced in-plane dimensions. Additionally, the MIS process realized a compact chip size of just 0.4 mm × 0.4 mm. Each of these approaches enabled the creation of an absolute pressure sensor chip from a single wafer, employing a procedure that encompasses small-hole etching, hole sealing, and piezoresistor doping/annealing. With these techniques, the thickness of the ultrathin diaphragm can be tailored via the etching parameters, resulting in reduced processing costs. However, the miniaturization of the pressure sensor also introduces challenges in wafer slicing and packaging. Fortunately, these hurdles have been overcome with the implementation of temporary bonding technology during thin-wafer processing.

In addition to fabrication, the design of sensors featuring ultrathin diaphragms warrants further investigation. Traditional piezoresistive pressure sensors have piezoresistors crafted through annealing in tubular furnaces, leading to a PN junction depth spread of >1 μm. Given that the pressure diaphragm typically measures <5 μm, this can lead to a notable reduction in sensitivity. Future research should prioritize the creation of highly stable, shallow junction piezoresistive fabrications, aiming for <0.3 μm, while also maintaining exceptional temperature stability performance. Many alternative sensing materials for microsized chips, silicon nanowires, graphene, etc., have also been proposed. Subsequent studies will likely shift focus toward methods for reducing nonlinearity and approaches for mass production. Microminiature pressure chips are typically employed in implantable pressure detection. Therefore, advancements in wireless packaging and signal transmission technologies will be of immense value, minimizing the inconvenience for patients.

### Leadless package pressure sensors

To reduce the size of pressure sensor packages and enhance reliability across various applications, researchers have introduced two leadless packaging techniques: a direct solder package founded on TSV/TGV and a sintering package built on a leadless pressure sensor.

Although TSV-based pressure chips have been incorporated into many miniaturized pressure sensors, several challenges remain. For instance, filled columns of copper or tungsten induce significant thermal stress under elevated temperatures, thereby compromising temperature stability. Consequently, forthcoming research should prioritize the development of partial-filling and all-silicon filling processes for sensors. This would address the issues stemming from the disparity in coefficients of thermal expansion, which lead to excessive thermal stress.

The sintering leadless packaging technique has been primarily tailored for high-temperature pressure sensors and has garnered significant interest over recent decades. These sintering methods notably enhance environmental adaptability and dynamic performance. To mitigate the impact of high-temperature sintering on the chip electrode, it is imperative to reduce the sintering temperature, which currently exceeds 500 °C. Additionally, elevated sintering temperatures result in increased sintering stress. Although the leadless packaging process has realized a more compact diameter, there remains a significant challenge in pulsation pressure sensing (e.g., package diameter <2 mm). In addition, an essential element that is currently lacking is a high-temperature compensation circuit, such as a high-temperature ASIC, which can significantly improve accuracy.

## Conclusion

After decades of development, pressure sensors based on microelectromechanical system (MEMS) technology have been widely adopted. The main progress and trends in the key fields are as follows:

1. In MDPS, a variety of structures have been introduced to enhance sensitivity by amplifying the stress concentration. Among these, the “diaphragm + island + peninsula” structure demonstrates superior sensitivity. However, the reported chip size is still quite large, which is not ideal in terms of cost-effectiveness and miniaturization objectives. Consequently, there is a need to further reduce the chip size by employing a slenderer diaphragm and a more superficial PN junction. With emerging medical needs, such as monitoring infant respiratory pressure, there is a pressing demand for even more sensitive pressure sensors.
2. Significant advancements have been made in the realm of quartz and silicon RPS, with notable progress in accuracy, resolution, size, and *Q*-factor. However, high-pressure RPS and micropressure RPS have received less attention, posing substantial design challenges. Additionally, there is a pressing need to enhance the frequency response of RPS. Currently, the frequency response of RPS is larger than 100 ms, restricting its application in high-dynamic-pressure measurements.
3. Regarding integrated pressure sensors, various combinations of pressure + “*x*” sensors have emerged, boasting impressively compact dimensions. These chips execute real-time, in situ temperature compensation and multiparameter measurements. Nonetheless, the interplay and decoupling mechanisms between measured parameters remain ambiguous, necessitating further investigation to enhance accuracy.
4. To minimize pressure sensor chips, various fabrication techniques, such as APSM, SoN, and MIS, have been introduced by researchers, substantially reducing both the in-plane dimensions and thickness of the sensor chips. For even more stringent size constraints, such as in thinner blood vessels, further innovations in miniaturized sensor-chip fabrication and packaging methods are needed. Concurrently, emerging 2D materials and nanoscale sensing materials, such as silicon nanowires, hold promise for microlevel sensor chips. However, the accuracy of these materials should be improved.
5. In the realm of leadless pressure sensors, FC-packaged sensors have been effectively integrated into industrial and consumer electronics, significantly advancing sensor package miniaturization. Nonetheless, the thermal stress from packaging still requires further mitigation. To accommodate leadless sensor packaging at elevated temperatures and ensure media compatibility, integrated sintering pressure sensors have been designed and refined, primarily leveraging glass and silver paste for sealing and connection. However, sensors utilizing the sintering approach generally demand larger chips, calling for size optimizations in future studies.

In this review, the main MEMS pressure sensors with new trends are analyzed and summarized, including their breakthroughs, problems, and probable solutions. Due to space constraints, certain MEMS pressure sensor categories were not fully covered. Therefore, these analyses will be continued in a subsequent special subject review.
